# Genome–host association mapping reveals wheat pathogen genes involved in host specialization

**DOI:** 10.1038/s41477-026-02269-w

**Published:** 2026-04-15

**Authors:** Cécile Lorrain, Alice Feurtey, Julien Alassimone, Bruce A. McDonald

**Affiliations:** 1 Plant Pathology Group, Institute of Integrative Biology, ETH Zurich, Zurich, Switzerland; 2 Present Address: Plant Protection—Phytopathology Group, Department of Crop Sciences, Research Institute of Organic Agriculture FiBL, Frick, Switzerland

**Keywords:** Genome-wide association studies, Agricultural genetics

## Abstract

Plant-pathogenic microorganisms, including the wheat fungal pathogen *Zymoseptoria tritici*, adapt to their host environment. In plants, genome-wide association studies (GWAS) have been extensively used to uncover the complexity of local adaptation and disease resistance. However, the application of GWAS to decipher the mechanisms underlying fungal pathogenicity and host adaptation trails far behind. Here we established a genome–host association approach to infer statistical associations between pathogen allele frequencies and host of origin for 832 fungal strains isolated from 12 different host cultivars during a natural field epidemic. We identified 2 to 20 genes associated with specialization to the different wheat cultivars, including one known effector gene, *Avr3D1*, as well as ten pathogenicity-related genes that provided a proof of concept for our genome–host association approach. Our study highlights the polygenic genetic architecture of host adaptation and provides a new application of GWAS in plant pathogens that transcends the limitations imposed by traditional phenotyping methods.

## Abstract (web summary)

This study introduces genome–host association (GHA), leveraging natural epidemics to map the genetic landscape of host adaptation in the pathogen *Zymoseptoria tritici*. Applied to 832 wheat fungal strains, GHA identified multiple genes underlying host specialization.

## Main

Plant-pathogenic fungi present a persistent menace to agricultural and woodland landscapes^1^. Fungal pathogens have evolved tailored mechanisms to promote pathogenicity and adapt to their hosts, using diverse molecules such as secreted effectors^2–4^ to subvert host defences and establish compatible interactions. A better understanding of the genetic basis of pathogen virulence can facilitate the characterization of host genetic factors associated with resistance or susceptibility^5^. Genome-wide association studies (GWAS) have increased our understanding of the genetic architecture governing adaptation in plant–pathogen interactions. Genome-wide association mapping is designed to identify candidate genes of interest by examining the statistical association between polymorphic genetic markers and phenotypes through comprehensive genome scans^6^. During the past decade, GWAS has been extensively used in plant pathosystems to unravel the complex genetic architecture of plant disease resistance phenotypes^7^. The use of GWAS to identify microbial pathogenicity factors lags far behind compared with research on plants^7^. In microorganisms, GWAS has primarily been used to discover genomic traits involved in pathogenicity and fungicide resistance, but these advances required prior knowledge regarding the host or fungicide phenotypes^8–15^. One reason for the relative lack of GWAS applications to plant-pathogenic microorganisms is the scarcity of extensive collections of genome-sequenced pathogen strains. A second reason is that GWAS in plant pathogens are performed primarily under highly controlled conditions, requiring time- and resource-consuming experiments that dramatically limit the throughput of in planta phenotypes^9,10^. Finally, recent advances such as co-GWAS, which jointly captures genotype-by-genotype interactions between hosts and pathogens^16^, have opened opportunities for studying coevolution. However, these approaches remain feasible only when large, well-matched host and pathogen genomic datasets are available.

In this study, we set out to scale up the discovery of virulence-related genes by acquiring pathogen traits directly under natural field conditions, thus circumventing the need for large-scale phenotyping experiments. We focused our efforts on the wheat pathogen *Zymoseptoria tritici*, the cause of the *Septoria tritici* blotch (STB) disease, which poses an important threat to global food security^17^. Managing STB is increasingly difficult due to the rapid evolution of *Z. tritici*, which quickly develops resistance to fungicides and overcomes key wheat resistance genes^18–20^. *Z. tritici* populations harbour all of the characteristics that facilitate rapid adaptation, including large effective population sizes, short generation times, high mutation rates and extensive gene flow among populations that encompass international agricultural landscapes^21^. Despite the importance of *Z. tritici* in agroecosystems, the majority of the avirulence genes associated with the 21 major isolate-specific STB resistance genes remain unknown, though three avirulence genes have been identified^5,9,14,22–25^. During the asymptomatic phase of its hemibiotrophic lifestyle, *Z. tritici* suppresses the activation of the host immune system^26,27^. A handful of the genetic factors underlying host immune suppression and infection promotion have been described in *Z. tritici*^14,28,29^. Adaptation to the host environment also involves interactions with the host-associated microbiota, which is known to shape the interaction between plants and fungal pathogens^6,26,30–32^. Studying these aspects of the *Z. tritici*–wheat interaction with GWAS requires large-scale phenotyping experiments limited by the resources allocated for in planta phenotyping in controlled conditions across large collections of strains.

Here we leveraged the power of GWAS to identify mechanisms of host adaptation in *Z. tritici* using natural wheat infections and whole-genome sequencing of 832 *Z. tritici* strains coming from 12 different host cultivars. We established a genome–host association (GHA) approach that associated pathogen allele frequencies with specific hosts by conducting a bootstrapped series of GWAS analyses. We performed 1,200 association mappings and identified 73 high-confidence loci involved in host specialization. We validated our GHA approach through the identification of a previously characterized virulence gene and by producing targeted gene knockouts for 16 candidate genes, with 10 confirmed to affect virulence. Our GHA approach should prove useful for high-throughput identification of genes associated with virulence and host specialization in other plant pathogens.

## Results

### GHA is a new approach for mapping the genetic landscape of *Z. tritici* host adaptation

**Fig. 1:**
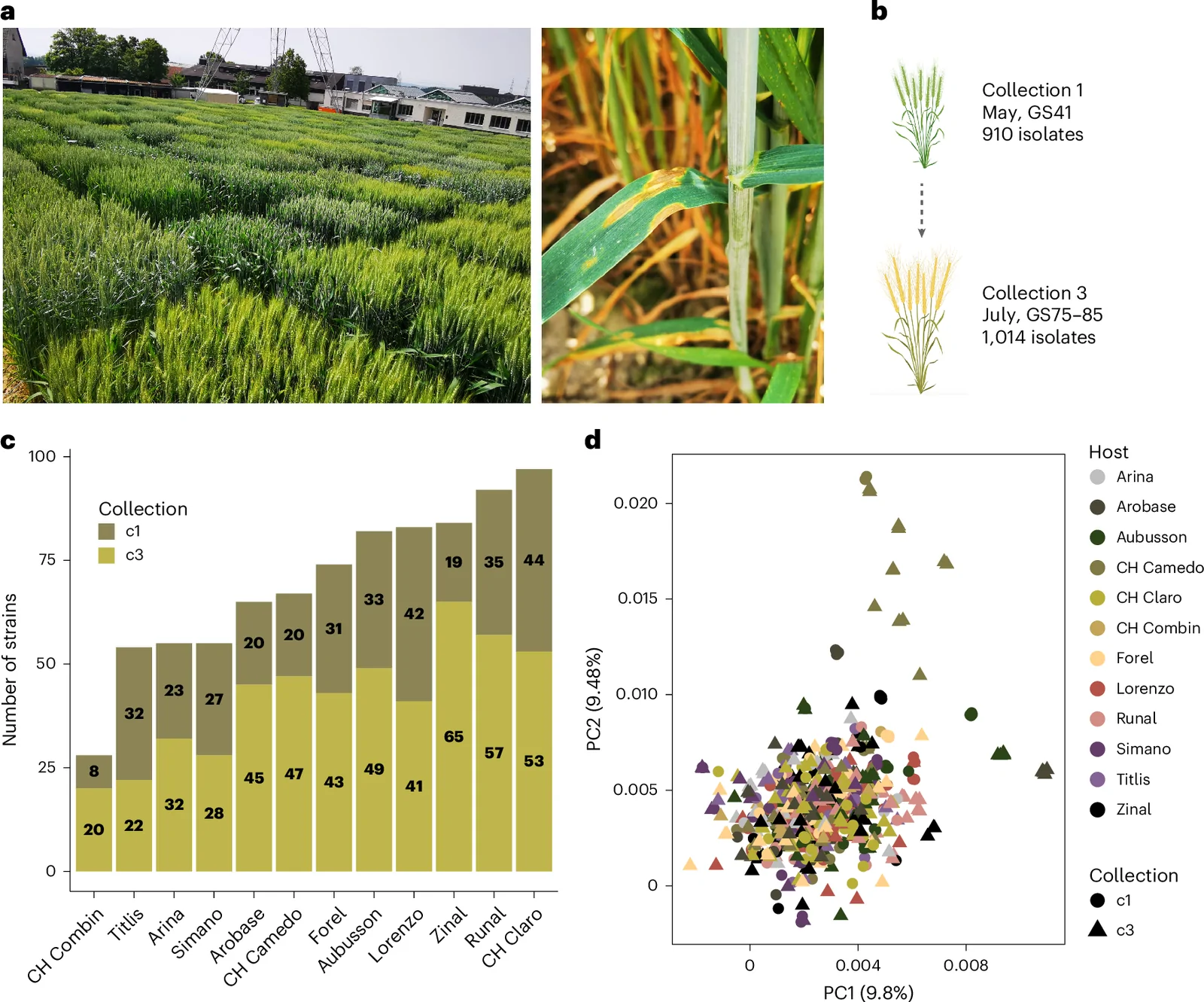
The 832 *Z. tritici* strains from a single field epidemic form one highly diverse genetic cluster. **a**, Sampling site at the Field Phenotyping Platform of the Eschikon Field Station of ETH Zurich, Switzerland (coordinates 47.449° N, 8.682° E), where 335 varieties from the GABI-wheat panel were grown in 2016 in two replicated fields and in plots of 2.02 m^2^ naturally infected by STB. A detailed map of the sampling scheme for the collection is shown in Extended Data Fig. 1b. **b**, Isolation of 1,924 *Z. tritici* samples during one epidemic season at different wheat growth stages (GS41 in May for collection 1 (c1) and GS75–85 in July for collection 3 (c3)). **c**, Number of strains per wheat cultivar and per time point in the final dataset of 832 sequenced strains. **d**, First and second principal components (PCs) from a principal component analysis based on biallelic variants in all strains. The colours and shapes indicate the wheat cultivar and collection time point of each strain. Illustrations in **b** created in BioRender; Lorrain, C. https://biorender.com/3auwqm5 (2026). Source data

**Extended Data Fig. 1:**
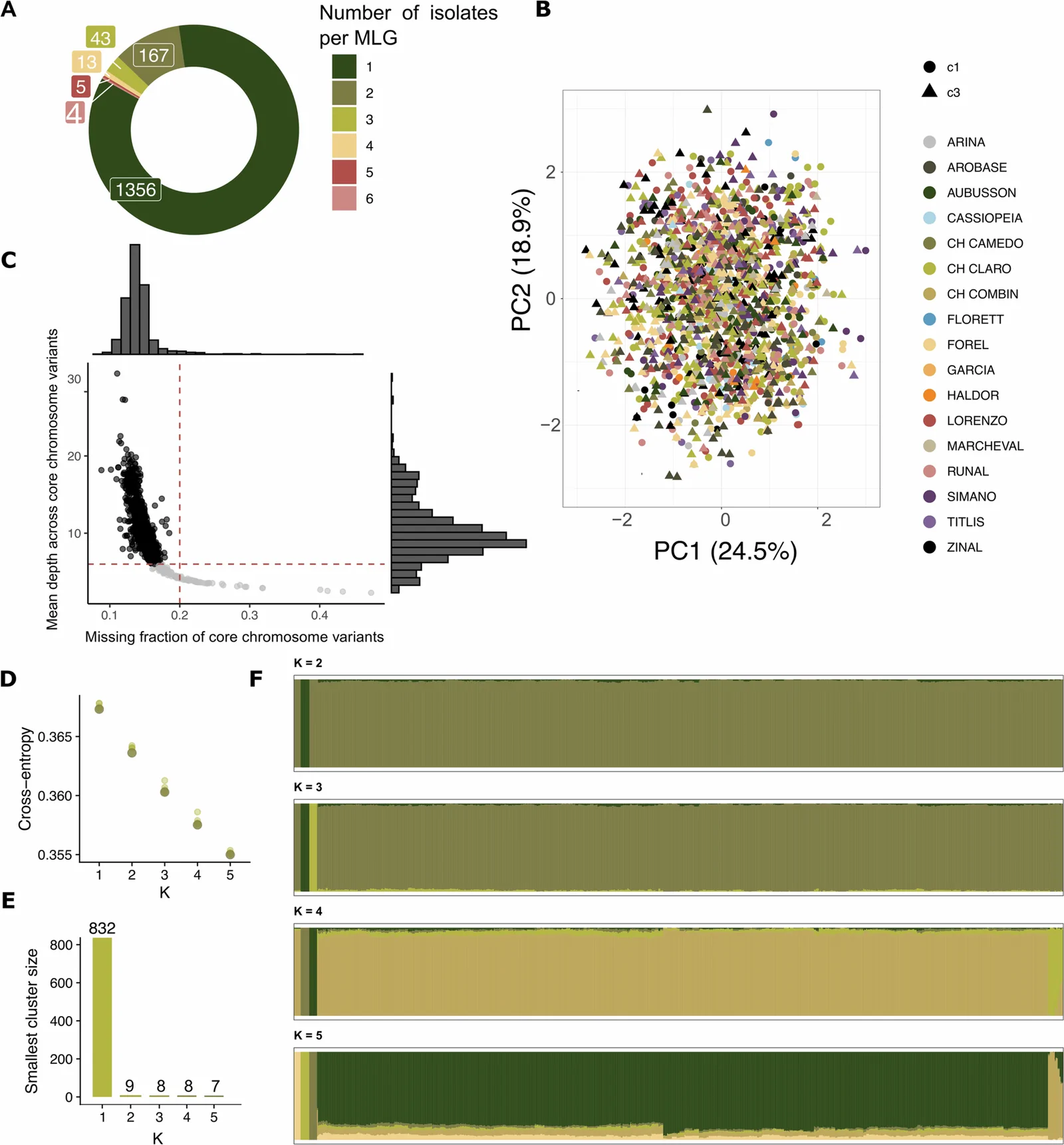
Clone correction, population structure, and genomic data filtering for GWAS. **A**) Frequency distribution of strains carrying particular multi-locus genotypes (MLGs) among the 1924 *Zymoseptoria tritici* isolates. Cases with two or more strains having the same MLG represent putative clones. **B**) First and second components of the principal component analysis based on MLGs. Colors and shapes describe the wheat cultivar and collection timepoint of each strain. **C**) Filtering of genome samples based on coverage and missing data. Samples with more than 20% of missing data (x-axis) and less than 6X of mean depth across core chromosomes (y-axis) were filtered out (light gray). **D**) Cross-entropy values for K = 1-10 inferred with the R package LEA^71^, used to select the optimal number of genetic clusters. Here K = 1. **E**) Size of the smallest inferred cluster for each K. **F**) Genetic clustering of the 832 *Z. tritici* strains for each K with each vertical bar representing one strain and colors corresponding groups. Source data

We assembled 1,924 *Z. tritici* isolates from a 2016 epidemic in Swiss wheat (May and July collections) sampled on 12 cultivars, identifying 1,588 multi-locus genotypes (MLGs) across 12 microsatellite markers with high genotype diversity^26^, which reflects the high genetic diversity of *Z. tritici* described in wheat fields^33^ (85.34% unique MLGs; Fig. 1, Supplementary Figs. 1 and 2, Extended Data Fig. 1 and Supplementary Tables 1–6). Whole-genome sequencing of 845 strains yielded 5,551,288 high-confidence variants across 832 samples, confirming the extensive genetic diversity of this population (Extended Data Fig. 1). Analysis of molecular variance, principal component analysis and population clustering confirmed that the 832 *Z. tritici* genotypes from this single-field population belong to a single genetic cluster (Fig. 1d and Extended Data Fig. 1), consistent with previous findings across Europe^21,34^. This lack of stratification supports the statistical power of GWAS by minimizing confounding and false positives, especially for complex traits such as host specialization^7^.

**Fig. 2:**
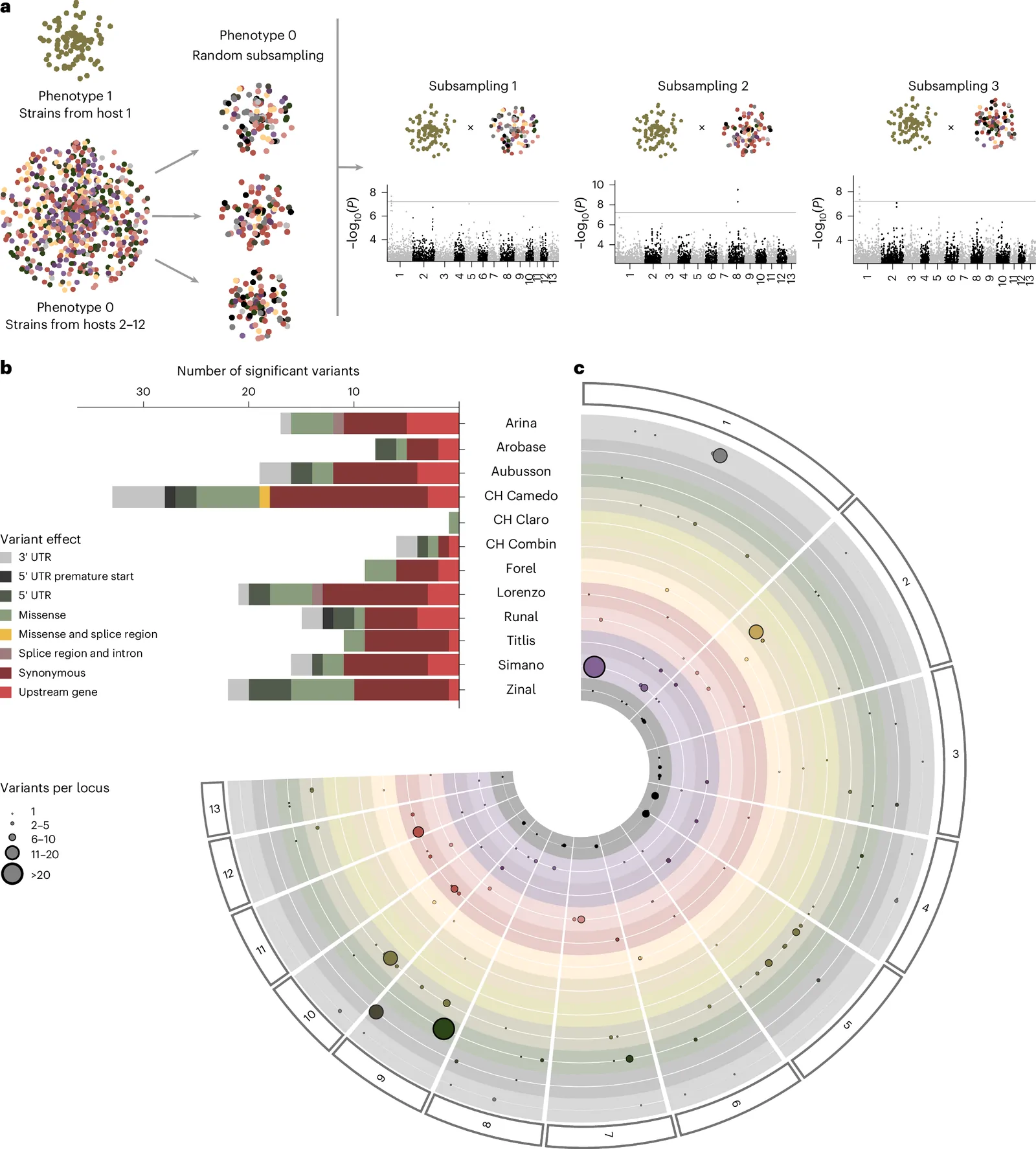
GHA approach in *Z. tritici*. **a**, Schematic representation of the GWAS approach comparing strains isolated from a focal wheat cultivar (phenotype 1) to a random subsample of strains coming from other host cultivars (phenotype 0), correcting for unbalanced group sizes with a bootstrapping approach^33,34^. The random subsampling and subsequent GWAS were repeated 100 times for each host. **b**, The number of significant variants and the predicted effect for each host cultivar. The *P* values for GWAS comparisons were obtained from a GEMMA univariate mixed linear model Wald test. Significant variants were corrected with the Bonferroni method by dividing 0.05 by the sum of unique variants analysed for each host. UTR, untranslated region. **c**, Fuji plot summary of genome-wide significant loci associated with the 12 host cultivars. Each layer represents one cultivar, and each dot represents the number of significant variants per locus. Source data

**Extended Data Fig. 2:**
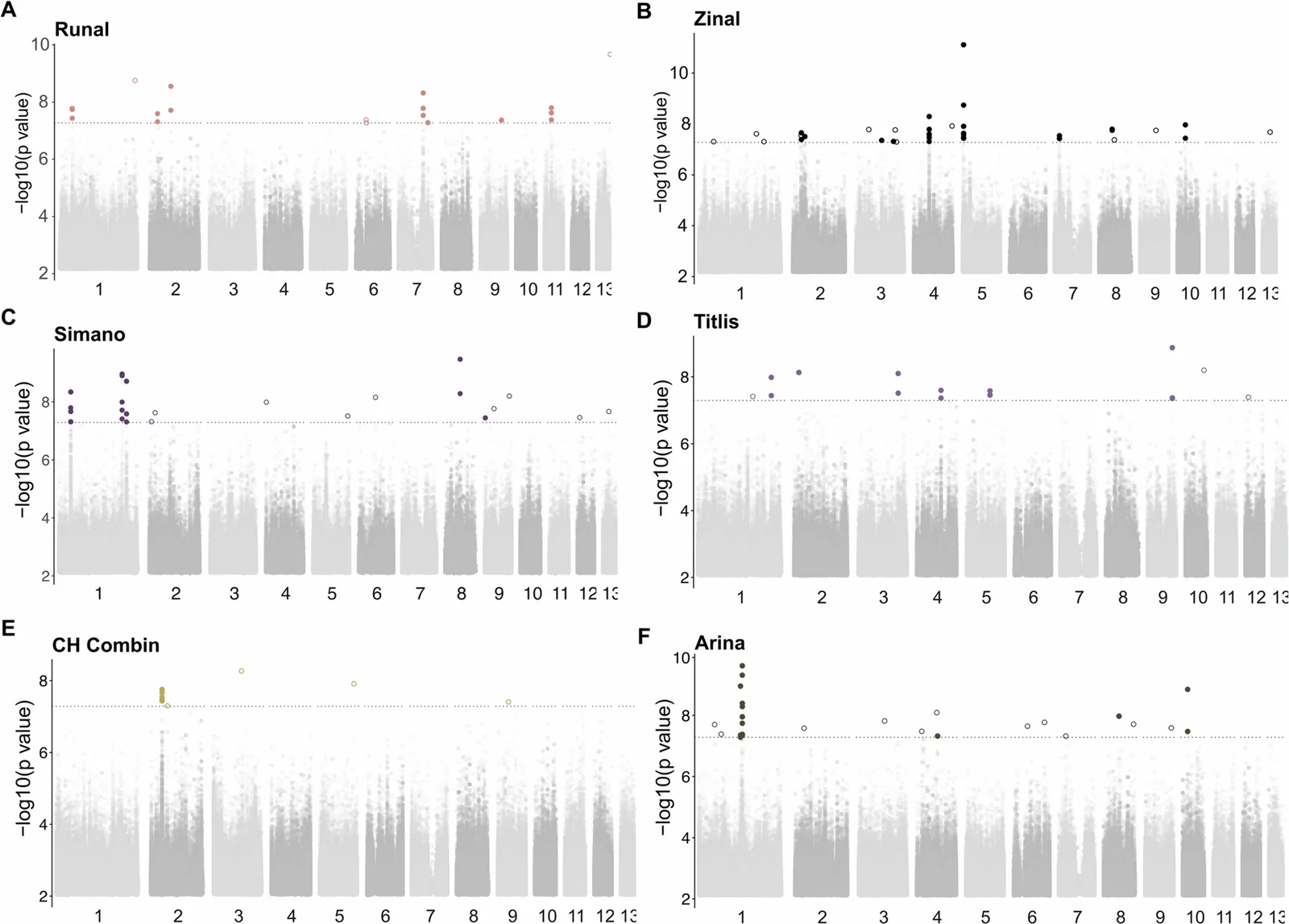
Manhattan plots for host-specific associated genomic traits for the most resistant wheat cultivars. **A**) Runal, **B**) Zinal, **C**) Simano, **D**) Titlis, **E**) CH Combin, and **F**) Arina. The colored points represent the significant variants. Circled points represent singleton significant variants. The horizontal line indicates the Bonferroni-corrected genome-wide significance threshold (α = 0.05). Source data

**Extended Data Fig. 3:**
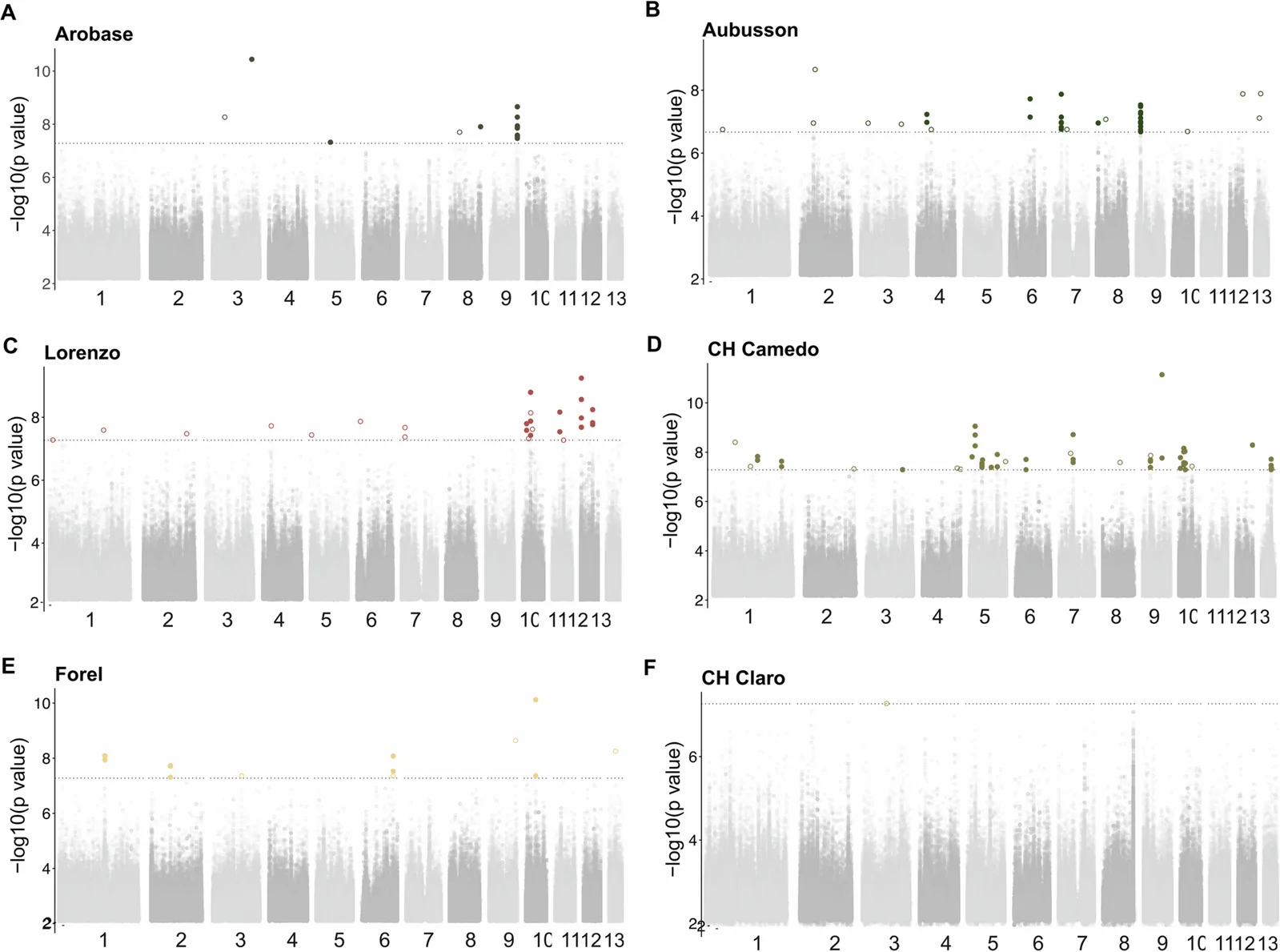
Manhattan plots for host-specific associated genomic traits for the most susceptible wheat cultivars. **A**) Arobase, **B**) Aubusson, **C**) Lorenzo, **D**) CH Camedo, **E**) Forel, and **F**) CH Claro. The colored points represent the significant variants. Circled points represent singleton significant variants. The horizontal line indicates the Bonferroni-corrected genome-wide significance threshold (α = 0.05). Source data

**Extended Data Fig. 4:**
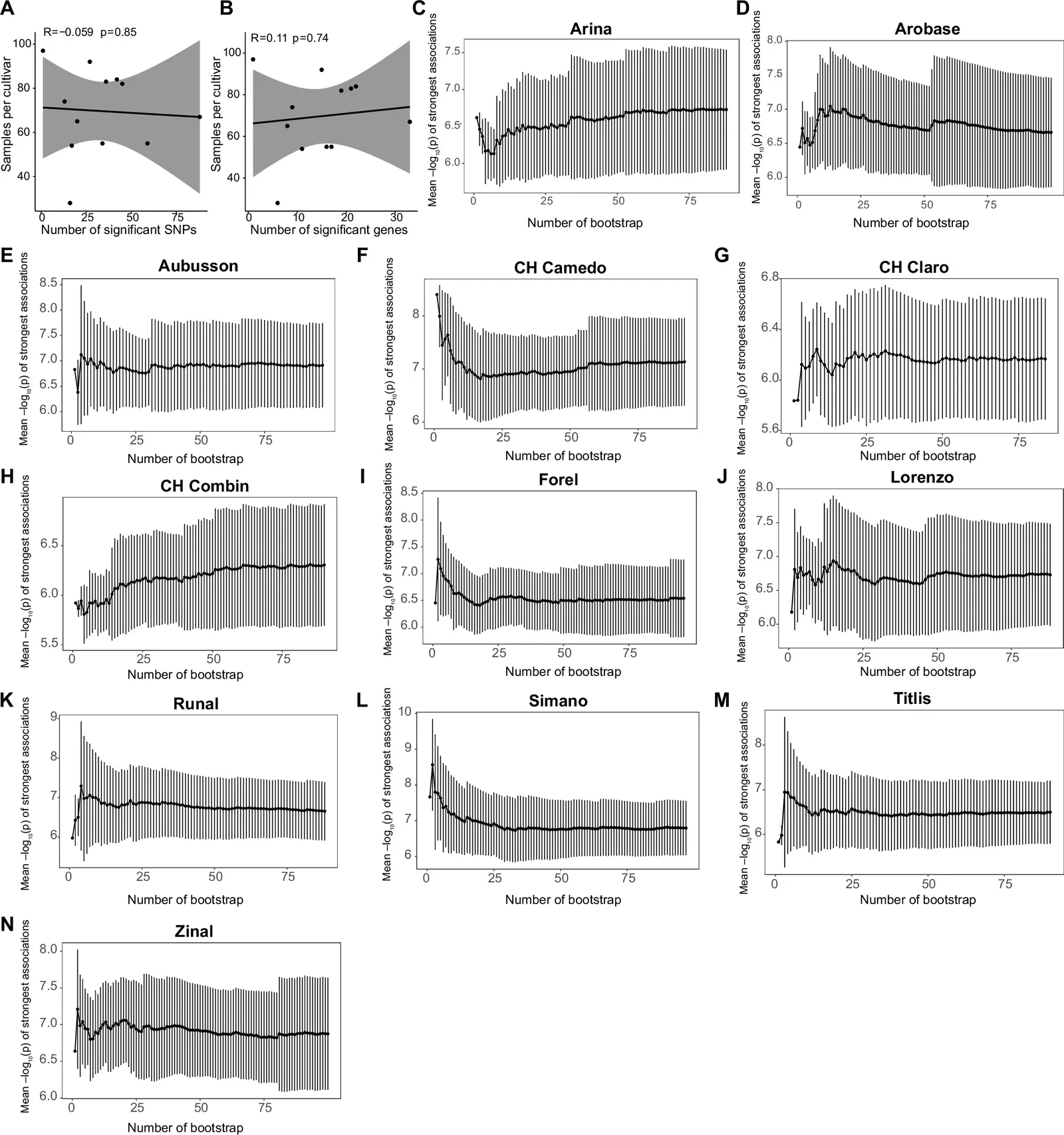
Lack of correlation between sample size and number of GHA associations per wheat cultivar. **A**) Relationship between the number of isolates per cultivar and the number of significant SNPs identified in the GHA analysis. **B**) Relationship between the number of isolates per cultivar and the number of significantly associated genes. Linear regression lines with 95% confidence intervals (shaded area) are shown. No significant correlations were detected, indicating that variation in sample size did not drive the number of associations recovered per cultivar (Pearson’s R and p-values shown on top of each plot). (**C**–**N**) Convergence curves showing the stability of the strongest association signal (minimum p-value) across increasing numbers of bootstrap replicates for each cultivar. For each value k (1–100), we computed the mean of the –log₁₀ of the minimum p-value obtained across the first bootstrap GWAS (black line), with vertical bars indicating the standard deviation across these k minima. For all cultivars, the cumulative mean stabilizes rapidly and additional bootstrap replicates contribute only minor changes, demonstrating that 100 iterations are sufficient to capture the reproducible association signal. Cultivars shown: Arina (**C**), Arobase (**D**), Aubusson (**E**), CH Camedo (**F**), CH Claro (**G**), CH Combin (**H**), Forel (**I**), Lorenzo (**J**), Runal (**K**), Simano (**L**), Titlis (**M**), and Zinal (**N**). Source data

**Extended Data Fig. 5:**
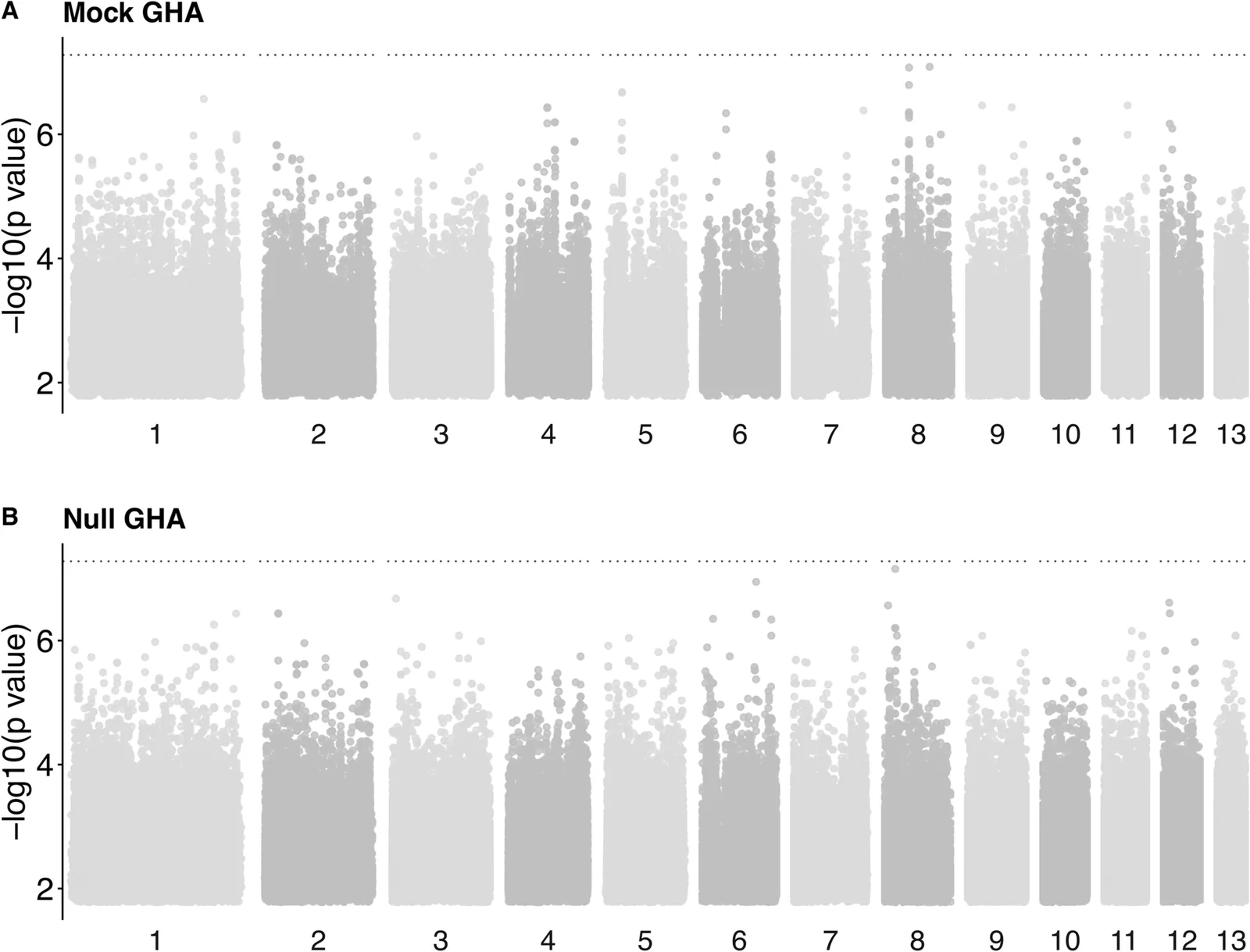
Negative-control genome–host association analyses (mock and null GHA) show no genome-wide significant associations. Manhattan plots from two negative-control GHA designs in which group membership was randomized while keeping the genotype dataset, SNP filtering, and GWAS model identical to the main analyses. **A**) Mock GHA: a random set of 80 isolates was designated as a pseudo ‘focal host’ test group and compared against the remainder of the collection. The procedure was repeated across 100 bootstrap iterations. **B**) Null GHA: two random groups of 80 isolates each were compared. The randomization of both groups was repeated across the 100 bootstrap iterations. The horizontal line indicates the Bonferroni-corrected genome-wide significance threshold (α = 0.05). Source data

To better understand the genetic landscape of *Z. tritici* host specificity, we performed a GHA, using GWAS to identify genetic variants associated with specific wheat cultivars. Briefly, our approach is as follows: for each of the 12 cultivars, we statistically compared allele frequencies in *Z. tritici* strains isolated from a focal cultivar with a random subsample of *Z. tritici* strains isolated from the other 11 cultivars. Our assumption was that alleles important for infection in the focal cultivar would be enriched in the strains collected in this cultivar when compared with the overall *Z. tritici* population in the same field. We corrected for unbalanced group sizes with a bootstrapping approach^35,36^; that is, the random subsampling was done 100 times, and each GWAS was performed with a linear mixed model and taking into account relatedness for each subsampling^37^, on variants with minor allele frequency higher than 5% and with less than 20% missing data (Fig. 2a). This resulted in 1,200 GWAS tests based on 928,182 to 978,139 variants (Supplementary Table 8). We identified 398 single nucleotide polymorphism (SNP) variants associated with 12 different wheat cultivars. The number of variants associated with host specialization on 11 of the cultivars ranged from 13 (Forel) to 88 (CH Camedo) after Bonferroni correction (*α* = 0.05; Fig. 2b,c, Extended Data Figs. 2 and 3 and Supplementary Table 9). Only one SNP was significantly associated with the cultivar CH Claro, but since it barely reached the Bonferroni correction threshold, we did not consider this locus further (Extended Data Fig. 3). We identified 158 genes with more than one overlapping or neighbouring significantly associated SNP, including 73 genes containing more than two significant SNPs in their sequences (Supplementary Table 9). We tested whether sampling depth influenced association detection by correlating the isolate count per cultivar with the number of significant SNPs or genes (Extended Data Fig. 4a,b). No significant relationship was found (*R*^2^ = −0.06 and 0.11; *P* = 0.85 and 0.74), suggesting that sample size had little effect on signal strength. Analysis of *P*-value stability across resampling rounds showed convergence after ~40–60 iterations, confirming that 100 resampling replicates were sufficient (Extended Data Fig. 4c–n). To estimate the rate of potential false-positive associations, we performed two negative-control analyses. In a ‘mock GHA’, we drew a random set of 80 genotypes as a pseudo-focal host group and performed GHA. In a ‘null GHA’, we compared two random groups of 80 genotypes and resampled these two groups between each bootstrap iteration. In both cases, no SNP reached the significance threshold (Extended Data Fig. 5). Our iterative resampling framework therefore successfully stabilized sample size disparities between cultivars, while identifying pathogen loci associated with specific hosts without direct phenotyping. It is worth noting that within-season spread of *Z. tritici* is largely asexual after epidemic onset^33,34^. Therefore, potential non-independence between time-point collections would mainly reflect clonal expansion, which we mitigated with clone correction and mixed-model relatedness correction. Denser per-cultivar sampling in future work could further enhance the resolution of time-stratified GHA. In this study, our locus-retention criteria favour sensitivity in the context of a likely polygenic architecture, and associations should therefore be interpreted as a prioritized repertoire of candidate loci.

The GHA approach shares the core assumptions of classical GWAS, including the need for sufficient genetic diversity, linkage disequilibrium between markers and causal variants, and appropriate correction for population structure^38^. As for any GWAS based on allele-frequency enrichment, the robustness of GHA can be influenced by sampling variance and epidemiological context, such that random over- or under-representation of alleles in a given cultivar-specific sample can contribute to apparent host-associated signals. In our study, the combination of equal-sized test/control groups, repeated bootstrap comparisons, mixed-model correction and locus prioritization was designed to limit such stochastic effects and support robust candidate discovery. At the conceptual level, GHA leverages host of origin as a proxy for the host background encountered by the pathogen during natural infection. In contrast to co-GWAS frameworks, which explicitly test reciprocal and genotype-by-genotype effects and offer high resolution of host–pathogen co-evolution^16^, GHA is designed for field settings where matched host genotypes, large numbers of different hosts and large-scale controlled phenotyping are not available. This enables high-throughput association studies under field conditions, where large-scale phenotyping is often impractical. Although this study focused on *Z. tritici*, whose high genetic diversity, frequent recombination and low clonality enhance the effectiveness of GHA^21,33^, it should still be broadly applicable to other microbial pathogens, as these criteria have also been met in other fungal species^39,40^. Similarly, association methods tailored to bacterial species, such as *k*-mer-based or pangenome-wide GWAS, have enabled the identification of virulence or host-specificity loci^35,41^. Finally, the principle of GHA is compatible with all types of genomic variation, including *k*-mers and structural variants, which can be particularly relevant in pathogens with dynamic accessory genomes. These features make GHA a flexible and scalable strategy for dissecting host specialization across a wide range of pathosystems.

### GHA recovers a known avirulence factor and pinpoints ten pathogenicity-related genes

To validate the GHA approach, we assessed its ability to identify pathogenicity-related genes. Among the 73 high-confidence GHA loci, 48 genes (65%) were annotated to encode diverse functions, including functions that would be expected to affect host specificity, such as predicted effectors (4 genes), transcription factors (3 genes), plant-cell-wall-degrading enzymes and peptidases (4 genes) and proteins containing kinase domains (3 genes) expected to affect regulatory processes (Supplementary Table 9)^42^. Our GHA identified a wide array of functions likely to be associated with host specialization under natural field conditions, which is consistent with the complex genetic architecture of host specialization in the *Z. tritici*–wheat pathosystem^15,43,44^. In addition, these results illustrate that molecular plant–microbe interactions extend far beyond the canonical gene-for-gene model of effector-triggered immune responses leading to host resistance^45,46^ and probably involve a wide range of molecular functions that contribute to promoting pathogen infection and adaptation to a specific host environment^1,2,47,48^. In plants, GWAS has confirmed the polygenic and dynamic genetic basis of disease resistance^7^. Here we depict a similar picture of a polygenic host adaptation that occurs in fungal pathogens.

**Extended Data Fig. 6:**
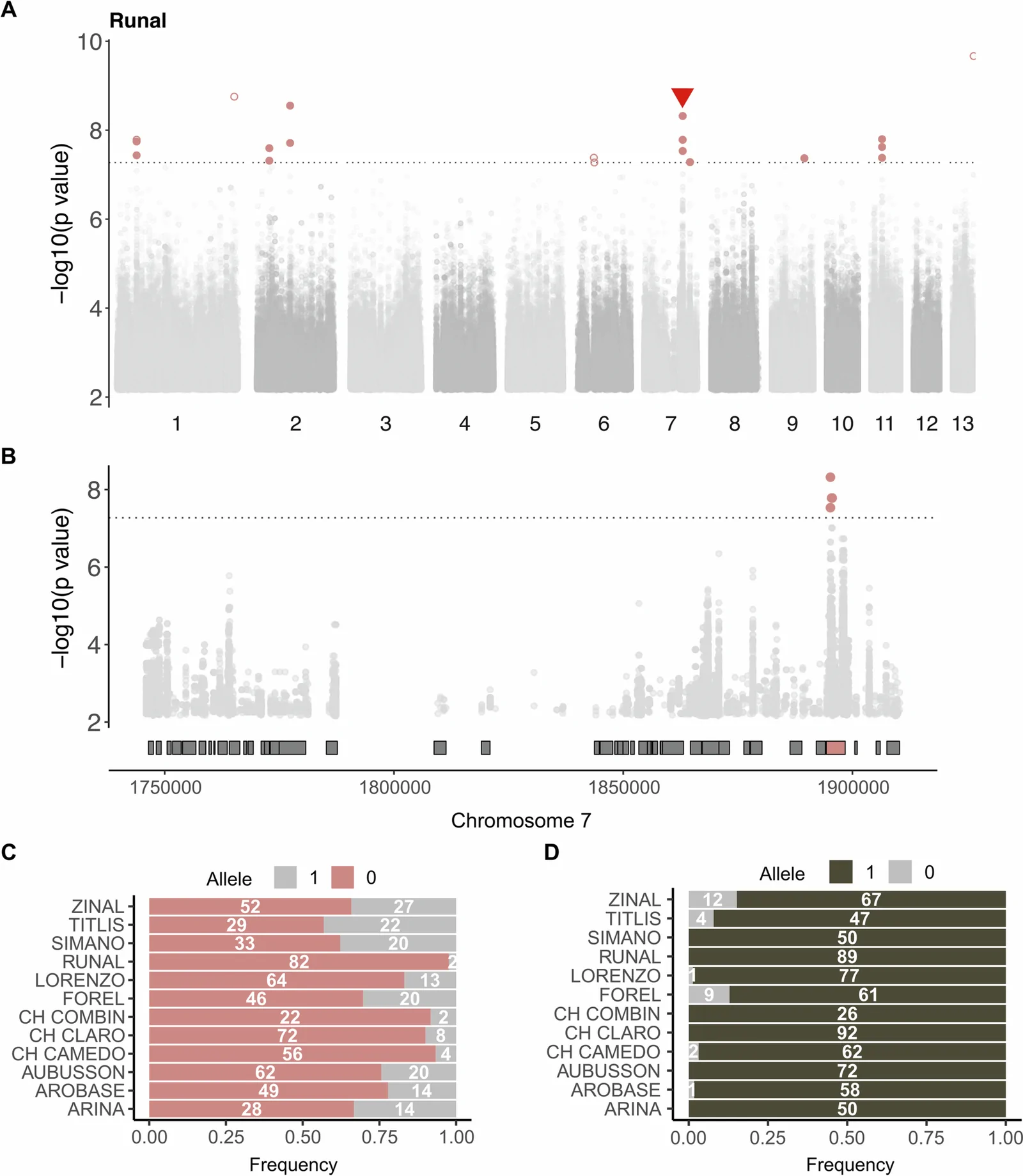
GWAS-identified the known effector gene *Avr3D1*^20^ that was specifically associated with cv. Runal but not *AvrStb6*. **A**) Manhattan plot for the GWAS related to Runal specificity, the colored points highlight the significant variants found on chromosome 7. **B**) Close-up Manhattan plot (top panel) on the previously identified quantitative trait locus (QTL) mapping from Stewart et al. (2018)^36^. The bottom chart represents the >30 genes of the reference genome IPO323^51^ found in the QTL region with the Avr3D1 gene represented by a pink box, other genes by grey boxes. **C**) Allele distribution of the most significant variant in Avr3D1 in the entire population according to host. Alternative allele (allele 1) in grey and reference allele (allele 0) in pink. **D**) Allele distribution of the most significant variant in AvrStb6 from Zhong et al., (2017)^9^ in the entire population according to host. Alternative allele (allele 1) in black and reference allele (allele 0) in grey. Source data

In addition to identifying genes with diverse functions, we anticipate that our approach will recover classical effectors, including previously characterized *Z. tritici* avirulence factors. In the cultivar Runal, quantitative resistance to Swiss strains has already been demonstrated to involve a gene-for-gene interaction, including one avirulence factor and a major resistance gene^24,44,49^. Consistent with these findings, our GHA performed using *Z. tritici* strains sampled from Runal identified a significantly associated locus that encompassed the recently reported avirulence gene *Avr3D1* (Extended Data Fig. 6)^24,44,49^. This finding illustrates that our GHA approach identifies genes involved in host specialization. In contrast, we did not identify a significant association at the *AvrStb6* gene locus, despite including the cultivar Arina, which is known to carry both the *Stb6* and *Stb15* resistance genes^22,25^. Our approach identifies allelic enrichment in strains isolated from specific host cultivars and thus depends on genetic variation at the locus of interest as well as the presence of both virulent and avirulent strains. An allele frequency analysis of the top *AvrStb6*-associated SNP reported by Zhong et al.^9^ revealed that nearly all the strains from all 12 wheat cultivars carried the same allele (Extended Data Fig. 6d). These findings are consistent with recent work that reported a complete absence of the avirulent AvrStb6 isoform among modern *Z. tritici* isolates and showed that a single virulence isoform has become globally predominant^50^. This apparent fixation of the virulent *AvrStb6* allele probably results from strong historical selection imposed by the widespread deployment of *Stb6* in commercial wheat^23,51^. Furthermore, the persistence of the virulent allele even in cultivars that may still carry the susceptible *Stb6* allele could suggest that it does not impose a detectable fitness cost on the pathogen. One limitation of our current design is the lack of information on the specific resistance genes present in each wheat cultivar, which hinders direct analysis of pathogen genotype × host resistance gene interactions. Future studies could address this by using cultivars with well-characterized and contrasting resistance gene profiles, enabling targeted detection of specific associations.

**Extended Data Fig. 7:**
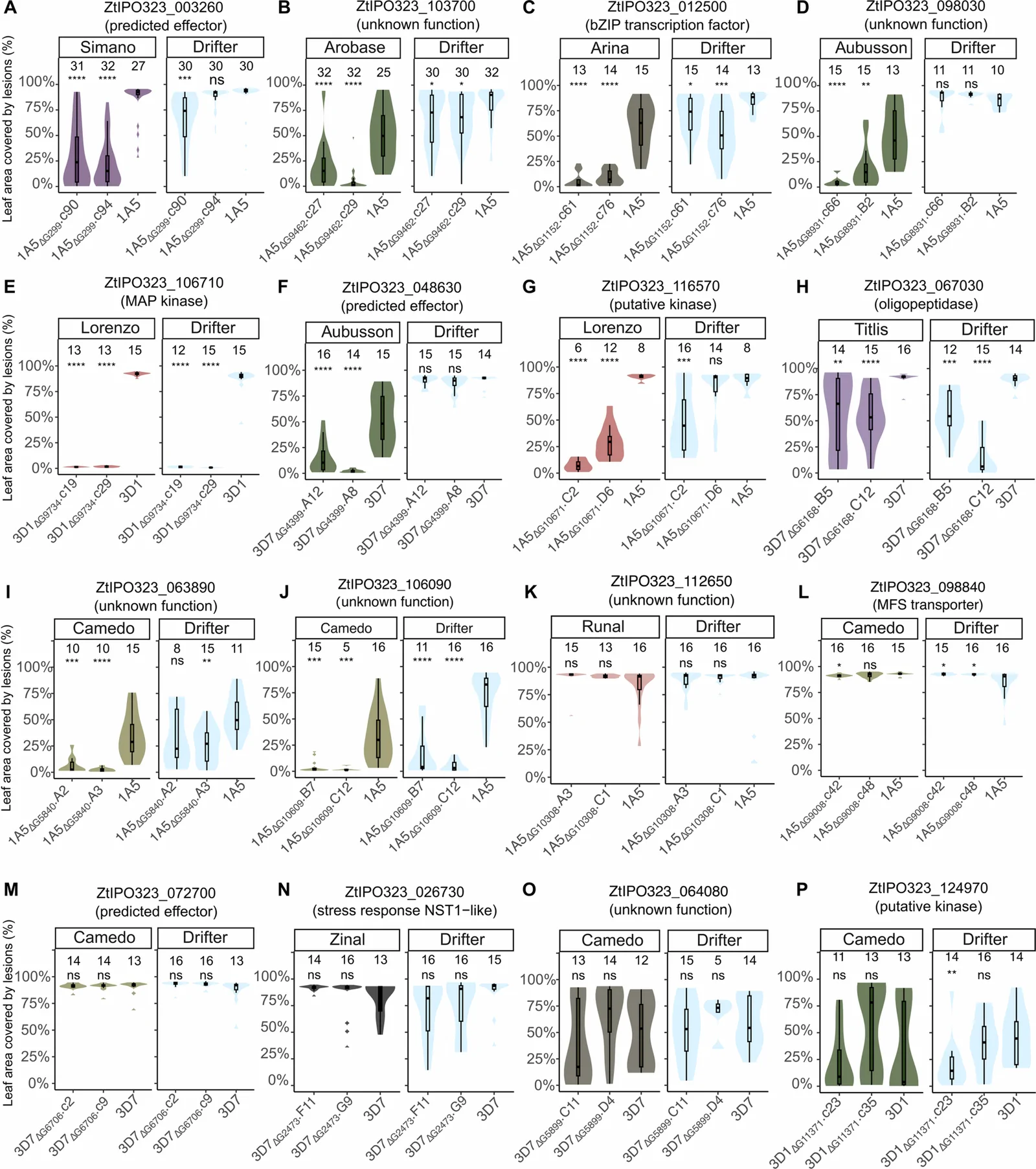
*In planta* virulence phenotypes of *Zymoseptoria tritici* gene deletion lines for 16 GHA-prioritized candidate loci. **A**–**J**) Gene deletions causing a significant reduction in virulence compared to the wild-type strain on at least one wheat cultivar. **K**–**P**) Gene deletions that did not significantly affect virulence *in planta*. The percentage of leaf area covered by lesions at 21 days post-inoculation (dpi) on the GHA-focal and susceptible (Drifter) wheat cultivars, following infection with *Z. tritici* deletion mutants and wild-type strains. Each violin plot displays the distribution of percentage of leaf area covered by lesions values across individual infected leaves, with overlaid boxplots showing the median and interquartile range. Mutant lines were generated in a virulent genetic background (1A5, 3D1, or 3D7), depending on which strain carried the GHA-identified ‘virulent’ allele (that is, most frequent allele in the 2016 Swiss *Z. tritici* population used for GHA). The number of infected leaves per strain is indicated above each violin. The lower and upper hinges on the boxplots represent the first and third quartiles respectively. The whiskers extend to values within 1.5 times the inter-quartile range from the quartiles. The central horizontal line shows the median. Significance between means of cultivar leaf area covered by lesions was tested with two-sides Wilcoxon test relative to the wild-type (ns = not significant; * = p < 0.05; **= p < 0.01; ***= p < 0.001; ****= p < 0.0001). Source data

**Extended Data Fig. 8:**
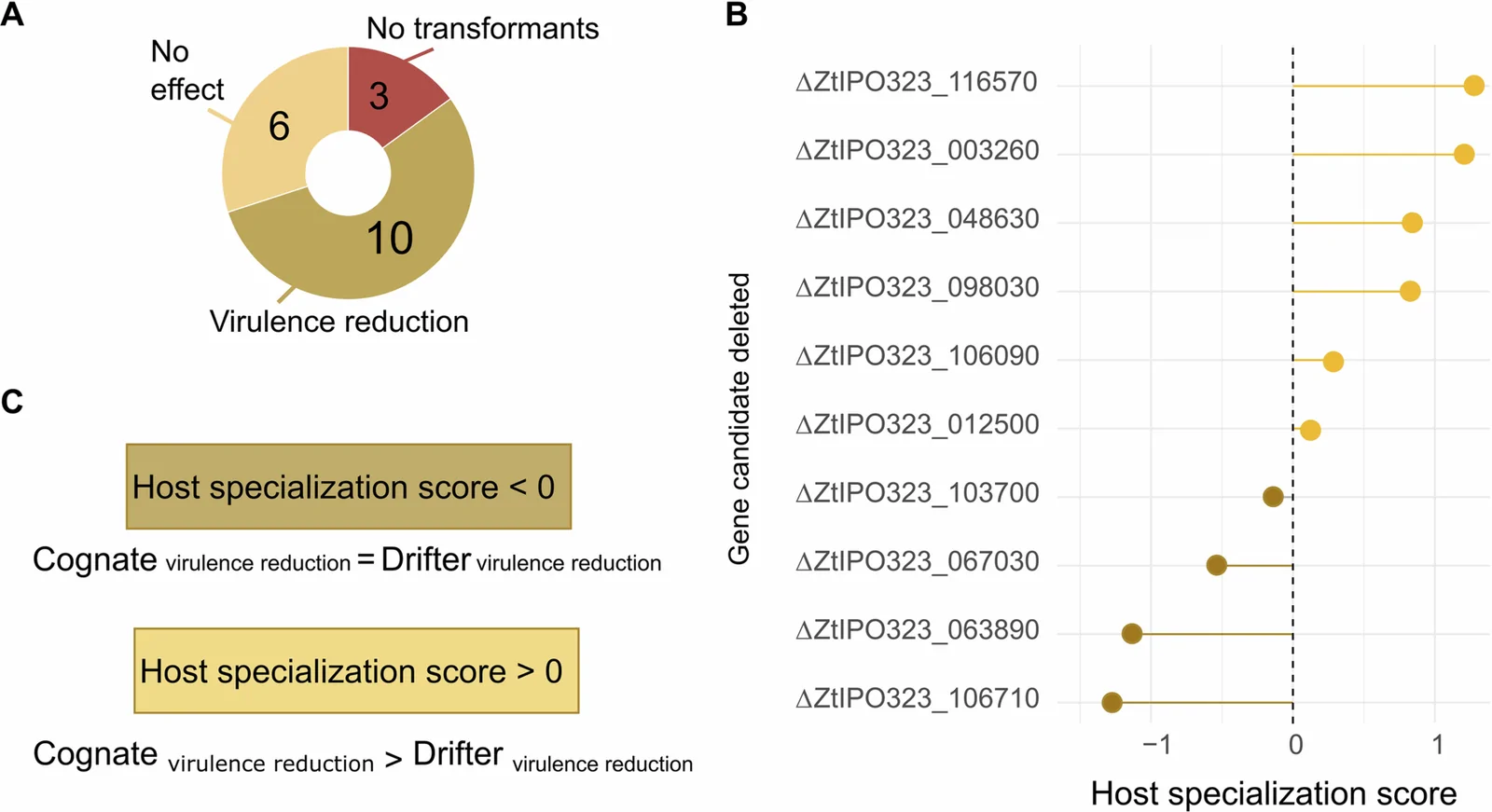
Identification of host-specialized and broad-spectrum pathogenicity-related genes. **A**) Summary of the gene deletion screen: among 19 candidate genes selected based on GHA significance and predicted function (for example, effector), 10 showed reduced virulence upon deletion, six showed no phenotypic difference with the wild-types, and three GHA-identified genes could not be deleted despite multiple attempts. **B**) Host specialization scores for the 10 gene deletions with a virulence reduction effect. The score was calculated as the difference in virulence reduction between the cognate cultivar (from which the association was detected) and the susceptible cultivar Drifter, relative to the wild-type background. **C**) Schematic representation of the host specialization score. Positive scores indicate a stronger virulence reduction on the cognate host (host-specific contribution), while negative scores indicate broader virulence effects Source data

To systematically evaluate the predictive power of GHA beyond known avirulence factors, we selected 19 candidate genes for functional validation through targeted gene deletion (Extended Data Figs. 7 and 8 and Supplementary Table 9). Candidates were prioritized on the basis of GHA significance scores, the number of non-synonymous SNPs and predicted functions. Each gene was deleted in a virulent reference strain (ST99CH_1A5, ST99CH_3D1 or ST99CH_3D7 (ref. ^52^)) carrying the predominant allele identified through GHA analysis, to test whether removing GHA-identified genes compromises virulence (Supplementary Table 9). We successfully generated deletion mutants for 16 of the 19 genes and phenotyped them on their cognate host cultivar as well as on a susceptible control cultivar (Extended Data Fig. 8a). Strikingly, 10 of the 16 successful deletion mutants (62.5%) exhibited significant virulence defects, demonstrating that GHA effectively identified pathogenicity-related loci associated with host specialization (Extended Data Fig. 7a–j). Six mutants showed no virulence differences under our experimental conditions (Extended Data Fig. 7k–p), which may reflect several non-exclusive possibilities. While we cannot rule out that some of these may be false positives from the GHA analysis, it is also possible that these genes exert more subtle effects on infection that were not captured in our assay. Notably, disease symptoms were evaluated at a late stage of infection, were optimized to detect strong virulence effects and were therefore less sensitive to specific infection stage dynamics^53^. Functional redundancy may also mask phenotypic consequences, a well-documented feature of fungal effectors^54^. Additionally, some genes may play roles under more complex field conditions, where environmental stresses and biotic interactions differ markedly from the controlled greenhouse environment, or during specific plant stages not evaluated in our seedling assays. Since GHA captures natural infection phenotypes, it is likely that some of the identified genes mediate interactions with other microbial taxa, with functional impacts in the native phyllosphere^55^. Together, this illustrates the broad potential of the GHA approach to uncover genes involved in diverse host–microbe interaction processes under natural conditions, offering opportunities to explore context-dependent pathogenicity.

### Functional validation of GHA candidates confirms the role of six genes in host specialization

To further dissect the genetic basis of host adaptation in *Z. tritici*, we classified GHA-identified genes according to the degree of host specialization observed in functional assays. By quantifying virulence reduction relative to the wild type on both cognate and control cultivars, we uncovered two distinct classes: host-specialized and broad-spectrum pathogenicity-related candidate genes (Extended Data Fig. 8 and Supplementary Table 9). Six deletions resulted in clear cultivar-specific virulence defects, suggesting a targeted role in overcoming host-specific barriers. These included genes encoding predicted effectors, a bZIP transcription factor (cultivar Arina) and a putative kinase (cultivar Lorenzo), supporting the hypothesis that diverse molecular mechanisms can underlie host specialization (Extended Data Figs. 7 and 8 and Supplementary Table 9). In contrast, four deletions affected virulence similarly across both control and cognate cultivars, indicating broader roles in pathogenesis (Extended Data Fig. 7 and Supplementary Table 9). We did not observe a consistent link between gene function and host specialization. For instance, genes of unknown function, signalling proteins and enzymes appeared in both host-specialized and broad-spectrum categories (Extended Data Figs. 7 and 8 and Supplementary Table 9). This functional heterogeneity reinforces the emerging view that host adaptation in *Z. tritici* is not confined to canonical effectors or known virulence factors but instead reflects a complex, polygenic architecture shaped by diverse biological processes.

**Fig. 3:**
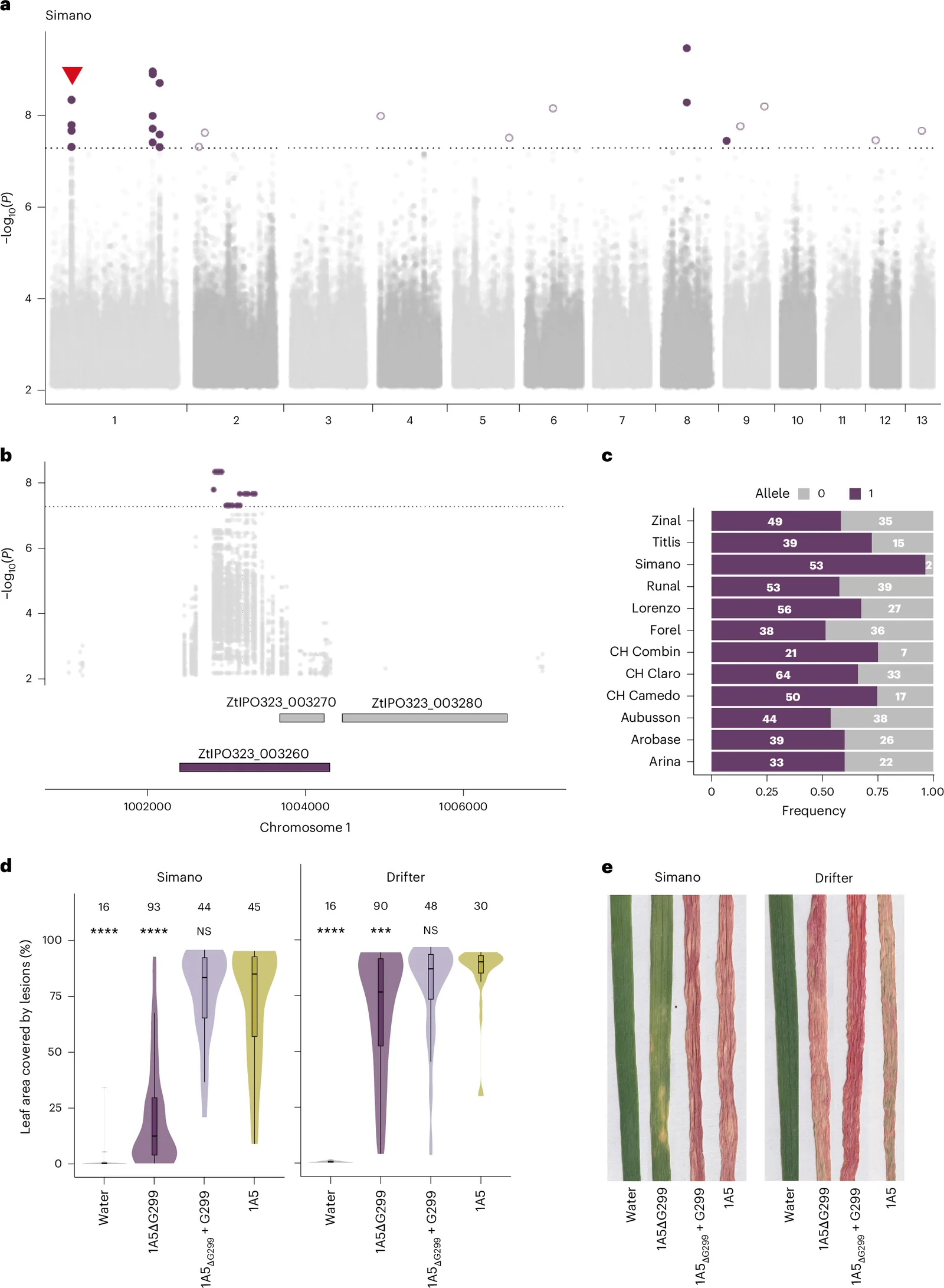
A GHA-identified effector gene required for cultivar Simano pathogenicity in *Z. tritici*. **a**, Manhattan plot of the associations between strains from Simano and other wheat genotypes, displaying the top 1% of values obtained from the 100 bootstrap GWAS. The horizontal dotted line indicates the Bonferroni threshold. The coloured circles represent the significant variants, with filled circles for loci with more than two variants and empty circles for singleton significant variants. The *P* values for GWAS comparisons were obtained from a GEMMA univariate mixed linear model Wald test. Significant variants were corrected with the Bonferroni method by dividing 0.05 by the sum of unique variants. The red triangle points the GWAS peak with the highest number of significant SNPs. **b**, Close-up view of the GWAS peak found on the chromosome 1 locus. The gene annotations on the reference genome IPO323 are shown at the bottom, with the candidate gene represented by a purple box and other genes by grey boxes. The *P* values for GWAS comparisons were obtained from a GEMMA univariate mixed linear model Wald test. Significant variants were corrected with the Bonferroni method by dividing 0.05 by the sum of unique variants. **c**, Allele distribution of the most significant variant in the entire population according to host. The alternative alleles (allele 1) are shown in purple and the reference alleles (allele 0) in grey. **d**, Percentage of leaf area covered by lesions of the wild-type virulent strain STCH99_1A5 naturally carrying the allele hypothesized to play a role in pathogenicity on Simano (1A5) as well as knockout and ectopic complementation lines phenotyped on Drifter and Simano. Mock-treated leaves were sprayed with water. The total number of wheat leaves harvested at 21 days post-infection is shown above each plot. The lower and upper hinges on the box plots represent the first and third quartiles, respectively. The whiskers extend to values within 1.5 times the interquartile range from the quartiles. The central horizontal line shows the median. Significance of the differences between means of cultivar leaf area covered by lesions was tested with two-sided Wilcoxon tests relative to the wild type (NS, not significant; ***P < 0.001; ****P < 0.0001). **e**, Representative pictures of wheat leaves covered by lesions at 21 days post-infection for the cultivars Drifter and Simano. Source data

**Extended Data Fig. 9:**
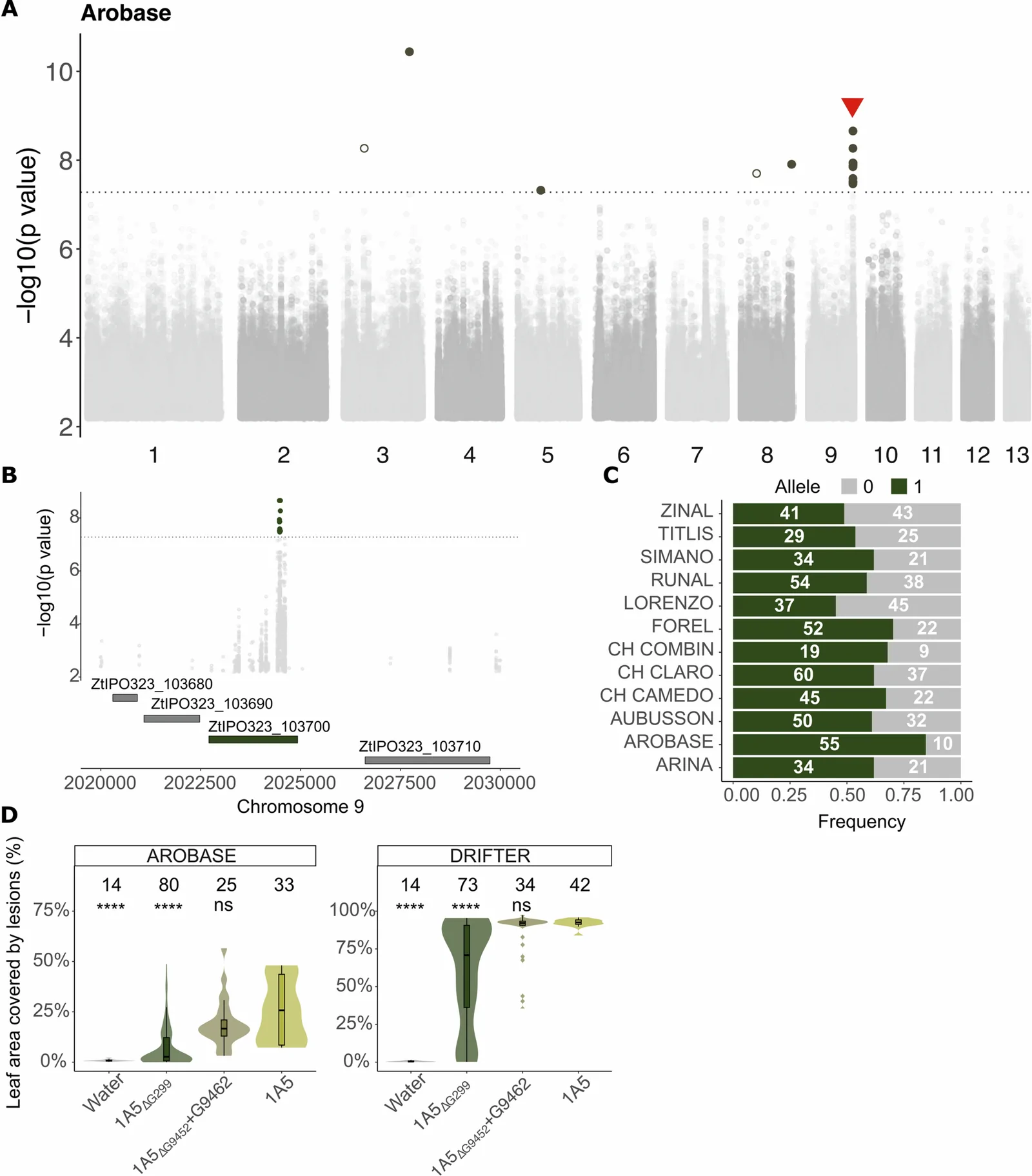
Identification and functional validation of a pathogenicity-related gene required for cultivar Arobase in *Zymoseptoria tritici*. **A**) Manhattan plot for associations between genotypes and presence on the cultivar Arobase displaying the top 1% of values obtained from the 100 bootstrap GWAS. The horizontal dashed line indicates the Bonferroni threshold. The colored dots represent the significant variants, filled circles for loci with more than two variants, and empty circles for singleton significant variants. **B**) Close-up view of the GWAS peak found on chromosome 9 (top panel) with the gene annotations on the reference genome IPO323 (bottom panel). The candidate gene used for functional validation is represented by a green box, other genes by grey boxes. **C**) Allele distribution of the most significant variant per host cultivar with the alternative allele (allele 1) in green and the reference allele (allele 0) in grey. **D**) Percentage of leaf area covered by lesions of the wild-type virulent strain STCH99_1A5 naturally carrying the allele hypothesized to play a role in pathogenicity on Arobase (1A5), knockout and ectopic complementation lines phenotyped on Drifter and Arobase. Mock-treated leaves were sprayed with water. The total number of wheat leaves harvested at 21 days post-infection is represented above each plot. The lower and upper hinges on the boxplots represent the first and third quartiles respectively. The whiskers extend to values within 1.5 times the inter-quartile range from the quartiles. The central horizontal line shows the median. Significance between means of cultivar leaf area covered by lesions was tested with two-sides Wilcoxon test relative to the wild-type (ns = not significant; * = p < 0.05; **= p < 0.01; ***= p < 0.001; ****= p < 0.0001). Source data

**Extended Data Fig. 10:**
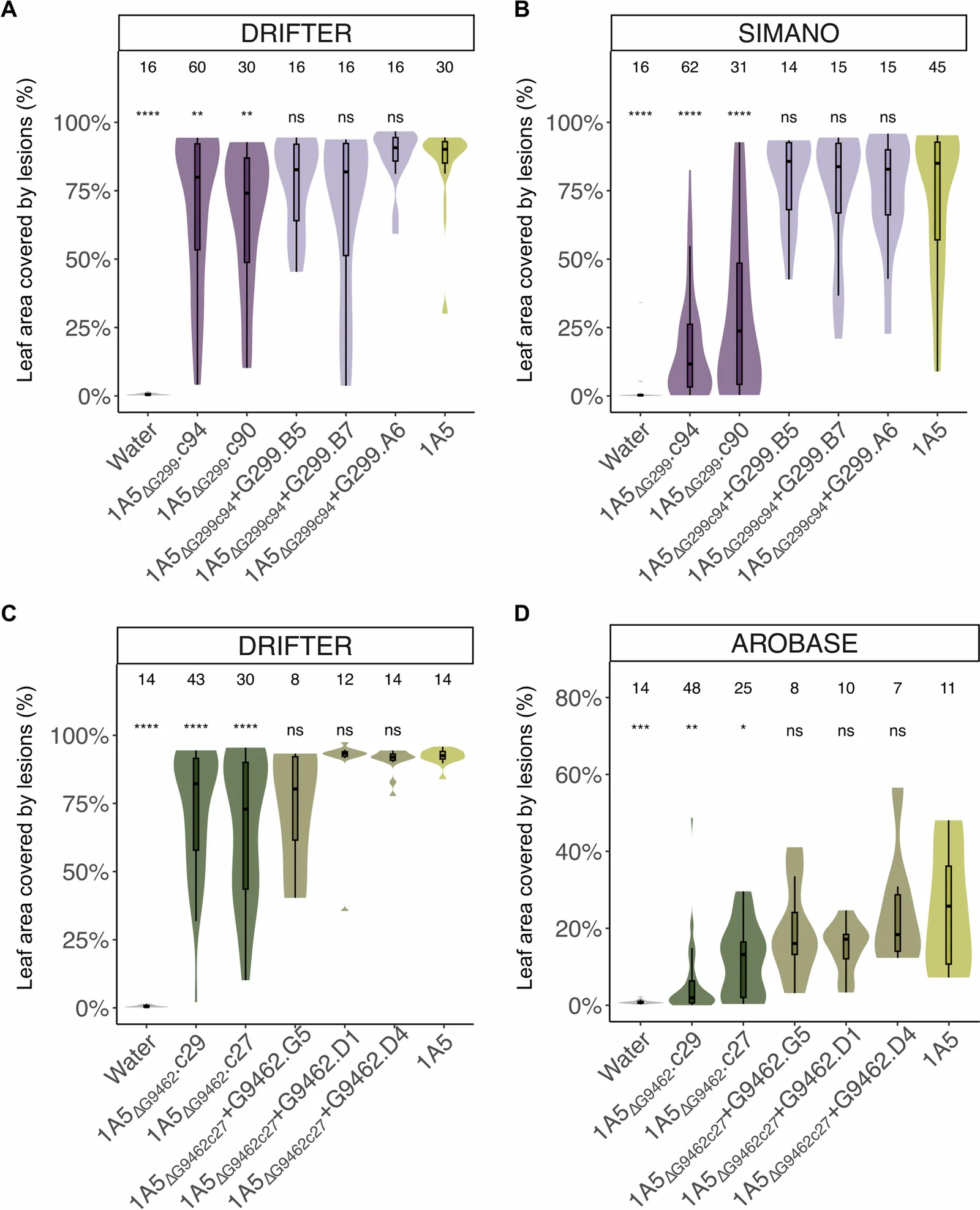
Functional validation of two candidate pathogenicity-related genes in *Zymoseptoria tritici* through independent gene knockout and complementation lines. Violin plots showing the percentage of leaf area covered by lesions (PLACL) at 21 days post-inoculation on wheat cultivars Drifter, Simano, and Arobase, following infection with the wild-type strain 1A5, independent deletion mutants (n = 2) of candidate gene STCH99_1A5G299 (**A**-**B**) identified for the cultivar Simano and STCH99_1A5G9446. (**C-D**) identified for the cultivar Arobase, and ectopic complementation lines (n = 3). The number of leaves per strain are indicated above each plot. Violin plots display the full distribution of PLACL values, overlaid with boxplots showing medians (central line). Water-sprayed negative controls are included. The lower and upper hinges on the boxplots represent the first and third quartiles respectively. The whiskers extend to values within 1.5 times the inter-quartile range from the quartiles. The central horizontal line shows the median. Significance between means of cultivar leaf area covered by lesions was tested with two-sides Wilcoxon test relative to the wild-type (ns = not significant; * = p < 0.05; **= p < 0.01; ***= p < 0.001; ****= p < 0.0001). Source data

We conducted a more in-depth functional validation for two pathogenicity-related candidate genes representing contrasting patterns of host specialization (Fig. 3 and Extended Data Figs. 9 and 10). We selected ZtIPO323_003260, a host-specialized predicted effector gene from Simano (host-specialization score, 1.2), one of the most resistant cultivars in our panel, and ZtIPO323_103700, a gene of unknown function from Arobase (host-specialization score, −0.1) exhibiting a minimal host-specific contribution and coming from one of the most susceptible cultivars in our panel (Fig. 3, Extended Data Figs. 8–10 and Supplementary Fig. 2). GHA mapping using Simano-derived strains revealed a strong host-specific association at ZtIPO323_003260, which encodes a 239-amino-acid predicted effector (Fig. 3a,b). Allele frequency analysis showed striking enrichment of the alternative ‘virulent’ allele in Simano-derived strains, present in 96% of strains from this cultivar (Fig. 3c and Supplementary Table 9). Conversely, GHA analysis using Arobase-derived strains identified ZtIPO323_103700, encoding a 740-amino-acid protein of unknown function (Extended Data Fig. 9a,b and Supplementary Table 9). The alternative allele at this locus showed enrichment in Arobase-derived strains (85% frequency; Extended Data Fig. 9c). We found that the enriched alternative allele was also present in strains found on other wheat cultivars, supporting the hypothesis that this allele may be generally advantageous for pathogenicity while being especially beneficial on specific hosts. We generated knockout and ectopic complementation lines in the virulent reference strain ST99CH_1A5, which carries the alternative alleles of ZtIPO323_003260 (that is, Zt1A5_G299 in ST99CH_1A5) and ZtIPO323_10370 (that is, Zt1A5_G9462 in ST99CH_1A5). In both cases, knockout lines (*n* = 2 independent lines per gene) displayed a significant decrease in virulence, while ectopic complementations (*n* = 3 independent lines per gene) restored the wild-type phenotypes after 21 days post-infection (Fig. 3d,e and Extended Data Figs. 9 and 10). These results confirm the causal roles for the two pathogenicity-related genes identified through GHA, with potential involvement in effector-triggered susceptibility^2^. Interestingly, the Arobase pathogenicity-related gene ZtIPO323_10370 encodes a protein of unknown function and does not carry a predicted secretion signal, which is needed to be defined as an effector^3,56^. Recent studies in the hemibiotroph *Pyricularia oryzae* have reported an unconventional secretion system that depends on codon usage and tRNA modification for the delivery of cytoplasmic effectors independently of the endoplasmic-reticulum–Golgi pathway^2,57^. Though this mechanism has yet to be described in *Z. tritici*, we did find orthologous genes corresponding to the Uba4–Urm1 system of *P. oryzae*^57^ in the functional annotations of IPO323 (that is, ZtIPO323_075920 and ZtIPO323_028380, respectively)^58^. This could suggest a similar unconventional secretion of cytoplasmic effectors in *Z. tritici*, which may partially explain why many non-secreted protein genes of unknown functions can be found in different *Z. tritici*–wheat association studies^13,15^. More in-depth functional characterization of these two genes will be needed to better understand how they promote *Z. tritici* infection.

## Conclusion

The GHA approach established here associates specific pathogen genetic variants with specific host cultivars, here using 832 *Z. tritici* strains sampled from 12 wheat cultivars that were exposed to the same natural epidemic. This method can provide a deeper understanding of the genetic basis of host specialization and presents a new application of GWAS to plant-associated microorganisms that transcends the limitations imposed by traditional phenotyping methods. The numerous loci identified in our GHA illustrate how a pathogen population infecting a single field can display extensive variation and develop a complex genetic architecture associated with host specialization. The identified genes encoded a diverse array of unknown functions as well as known functions, including plant-cell-wall-degrading enzymes, peptidases, secreted effectors, signal transduction pathways and transcriptional regulators. Our findings confirm previous descriptions of a vast reservoir of standing genetic variation existing within field populations^33^ and elucidate the complex genetic basis of *Z. tritici*–wheat interactions^9,13,15^. The two virulence-related genes we validated from our GHA approach provide a solid proof of concept for the method and represent a major step towards a better understanding of host adaptation mechanisms in a major wheat fungal pathogen.

## Methods

### Pathogen sampling strategy, genotyping and clone correction

We assembled a collection of 1,924 *Z. tritici* isolates sampled from a natural epidemic at the Field Phenotyping Platform of the Eschikon Field Station of ETH Zurich, Switzerland (coordinates 47.449° N, 8.682° E). Three hundred thirty-five varieties from the GABI wheat panel were grown in 2016 in plots of 2 m^2^ in two replicated fields^59,60^. *Z. tritici* strains were isolated from infected leaves of 12 wheat cultivars harvested on 20 May, 6 June and 4 July 2016. The 12 cultivars were chosen to reflect the observed range of STB quantitative resistance rankings reported in Karisto et al.^59^, on the basis of necrotic lesions (the percentage of leaf area covered by lesions (PLACL)) and pathogen reproduction (pycnidia density) (Extended Data Figs. 1 and 2). Briefly, the cultivar rankings were based on mean ranks derived from 21,420 infected leaves of 335 wheat varieties, where each leaf was scored for PLACL and pycnidia density^59^. The final scores reflect the average position of each cultivar across the full ranked leaf dataset, ranging from most resistant (lowest rank) to most susceptible (highest rank), for each trait separately. These rankings are provided here to contextualize the host panel and were not used as input for the GHA analyses, which relied on pathogen genotypes and host-of-origin labels.

Strains were isolated as described in Singh et al.^61^, by streak-plating of individual cirri on yeast malt sucrose agar amended with kanamycin (50 μg l^−1^). Single colonies were subsequently grown in yeast sucrose broth to produce blastospores that were used for long-term storage and DNA extractions. DNA extractions for both Illumina sequencing and microsatellite genotyping were performed from lyophilized blastospores grown for 7 days in yeast sucrose broth, using the QIAGEN DNeasy Plant Mini Kit (Qiagen). To clone-correct the 1,924 *Z. tritici* isolates for whole-genome sequencing, we genotyped each isolate using 12 neutral microsatellite markers in two multiplex PCRs^62^. Microsatellite amplification was performed using the QIAGEN Type-it Kit (Qiagen), and fragment analysis was performed using a 3730 DNA Analyzer (Applied Biosystems). Alleles were scored with the R package Fragman v1.0.9 (ref. ^63^), and MLGs were assigned using the poppr package v2.9.8 (ref. ^64^). We identified 149 alleles in total, with an average of 12.5 alleles per locus (Supplementary Tables 2–4)^62^. We found 1,588 MLGs, of which 85.34% (1,356) were found only once and 14.66% (232) at least two times (Extended Data Fig. 3a and Supplementary Table 5). The May and July collections showed a high and nearly identical genotype richness and a low abundance of clones, with a Simpson index (*λ*) of 0.998 and 0.999 and a genotype abundance (*E*) of 0.907 and 0.908, respectively (Supplementary Table 5). For collections from each wheat cultivar, the genotype abundance ranged from 0.85 (on Simano) to 0.97 (on Aubusson) (Supplementary Table 6). We performed an analysis of molecular variance to search for genetic differentiation between collection time points and hosts after clone correction and filtering out genotypes with too much missing data and with rare alleles (minor allele frequency <5%). We observed less than 1% of the molecular variance between collection time points (0.2% of variation) and between hosts within collection time points (0.3% of variation) (Extended Data Fig. 3b and Supplementary Table 7). The molecular variance increased to 4.4% between samples within hosts, but most molecular variance (95%) was found between samples (Supplementary Table 7). After multi-locus clone correction, we sequenced 845 strains sampled from 12 wheat cultivars exhibiting a wide range of quantitative resistance (Supplementary Table 1 and Extended Data Fig. 2).

### Genome sequencing, variant calling and population structure analysis

DNA libraries of 845 unique genotypes were sequenced with an Illumina NovaSeq6000 using 150-bp paired-end reads (Novogene Europe). An additional 144 *Z. tritici* strains from Singh et al.^61^, originating from the same collection but sequenced previously, were added to our dataset (NCBI SRA BioProject number PRJNA596434). Variant calling was performed using short-read mapping on the reference genome IPO323 (ref. ^58^). We trimmed reads with Trimmomatic v.0.35 (ref. ^65^), removing low-quality bases (<28), sequencing adapters and reads shorter than 50 bp for all sequencing samples. We mapped reads onto the reference assembly of IPO323 (ref. ^66^) using BWA v.0.7.17 (ref. ^67^) and removed duplicates with PICARD v.2.23.8 (https://broadinstitute.github.io/picard/). We called variants with GATK v.4.1.2.0 (ref. ^68^) using HaplotypeCaller with the option of ploidy 1. We merged single vcf with CombineGVCFs and GenotypeGVCFs with a maximum alternative allele number of three^68^. We filtered variants on the basis of the variant calling quality of FS > 10, MQ < 20, QD < 20, ReadPosRankSum between −2 and 2, MQRankSum between −2 and 2, BaseQRankSum between −2 and 2, and genotype depth lower than 3, as described in Feurtey et al.^21^. To remove errors in variant calling due to low sequencing depth, we filtered out any sample with more than 20% missing data and low depth coverage (<6 on core chromosomes) using vcftools v.0.1.16 (ref. ^69^). Finally, we removed clones that were not identified by multi-locus genotyping using the identity-by-state individual network with PLINK v.1.9.0 (ref. ^70^). Near clones were flagged by identity-by-state values higher than 0.997. We finally retained 832 filtered samples from the genomic dataset. We assessed potential population structure using a subset of linkage-disequilibrium-pruned, biallelic SNPs and filtered out missing data and minor allele frequencies <0.05 using PLINK v.1.9.0 (ref. ^70^). We then used the command pca to perform a principal component analysis to explore the distribution of genetic variation across our collection. Population structure was inferred using sparse non-negative matrix factorization as implemented in the R package LEA v3.22.0 (ref. ^71^). Sparse non-negative matrix factorization was run for *K* = 1 to 10 clusters, with five independent replicates per *K* using the default regularization settings and entropy minimization. Model fit was evaluated using the cross-entropy criterion, and for each *K*, the replicate with the lowest cross-entropy was retained. The optimal number of clusters was determined by identifying the point where increases in *K* yielded diminishing returns in cross-entropy reduction (*K* = 1). To assess robustness and avoid over-partitioning, we also examined the size of the smallest inferred cluster across *K* values. All analyses were performed on a linkage-disequilibrium-pruned dataset comprising 200,000 SNPs randomly sampled from core chromosomes.

### GHA study in *Z. tritici*

Genomic variants potentially associated with specific host types were identified using a GHA approach. SNP calling results from a dataset of 832 filtered strains were utilized. For each analysis, a test group consisting of strains sampled from a particular host was selected and compared against a control group of equal size. The control group was composed of randomly subsampled strains from the alternative host, excluding those present in the test group. The test group was attributed a phenotype of 1, and the control group was attributed a phenotype of 0 and added in a single phenotype CSV file.

We used the parser vcf2gwas v.0.8.7 (ref. ^37^) to perform GWAS of each test group against the control group. The vcf2gwas v.0.8.7 tool was employed to trim and filter biallelic SNPs pruned to remove variants in high physical linkage (1 SNP per kb) with bcftools^72^ and PLINK^70^, and convert the input VCF file while formatting the phenotype file to meet the requirements of GEMMA^73^. Relatedness matrices were calculated automatically with vcf2gwas v.0.8.7 for each bootstrap comparison to control for population substructure. The *P* values for GWAS comparisons were obtained from a GEMMA univariate mixed linear model Wald test, which evaluates the association of each SNP by testing the alternative hypothesis *β* ≠ 0 against the null hypothesis *β* = 0 and outputs the corresponding *P* values. This was performed independently for each bootstrap GWAS comparison. We corrected for multiple testing using the Bonferroni method by dividing 0.05 by the sum of SNPs analysed. We repeated the abovementioned process of comparing the test group against randomly subsampled control groups 100 times.

We performed 100 bootstrap GWAS per cultivar, as described by Epping et al. for host–microbe association studies using bootstrapping to correct for unbalanced sampling^36^. After conducting GHA for each cultivar sampling group, we combined all the associations and counted the total number of unique SNPs, counting each SNP only once, even if it appeared in multiple comparisons, evaluated across all bootstrap GWAS. This allowed us to apply a Bonferroni multiple testing correction, resulting in a specific Bonferroni threshold for each GHA. We considered a GHA-identified locus of interest if more than two SNPs were found significant within a 1-kb region or if the same SNP was significantly associated in more than two bootstraps. Finally, we prioritized the loci of interest according to their overlap with annotated genes and with a predicted non-synonymous substitution, using SnpEff^74^ to predict the effects of significant variants with a custom database based on the latest gene annotation of the reference genome IPO323 (ref. ^58^).

To evaluate whether variation in the total number of isolates per cultivar influenced the number of associations detected, we tested for correlations between sampling depth and (1) the number of significant SNPs and (2) the number of significant genes identified per cultivar. To evaluate the stability of association signals across bootstrap replicates, we extracted the *P* values obtained from each GWAS subsampling run per cultivar. To construct convergence curves, we calculated the cumulative mean and standard deviation of the −log_10_(*P*) values across increasing numbers of bootstrap replicates. Specifically, for each value of *k* from 1 to 100, we computed the mean and standard deviation of the first *k* minimum *P* values. The resulting curves depict how the average strength of the strongest association signal stabilizes as more replicates are added. Stabilization indicates that further bootstrap replications contribute little additional information.

Finally, we assessed false-positive associations by implementing two negative-control GHA designs in which phenotypes were randomized while keeping the genotype dataset, SNP filtering relatedness correction, mixed-model framework and Bonferroni correction identical to the main analyses. In the mock GHA, we randomly selected 80 isolates to form a ‘pseudo focal’ group independent of host of origin and compared them against the remaining isolates, repeating the full analysis for 100 bootstrap GWAS iterations (resampling the remaining 80 isolates each time). In the null GHA, we randomly resampled the two groups between each GWAS bootstrap (80 test versus 80 control) independent of host of origin. For both cases, no SNP reached significance thresholds.

### Functional validation of GHA-identified candidate genes

To experimentally validate the role of GHA-identified candidate genes in *Z. tritici* virulence, we performed targeted gene deletions in three virulent Swiss reference strains (ST99CH_1A5, ST99CH_3D1 and ST99CH_3D7)^52^, each carrying the predominant allele identified through the GHA analysis. Deletion mutants were successfully generated for 16 of these genes and phenotyped on both the cognate host cultivar and the susceptible control cultivar Drifter (Supplementary Table 9).

Gene knockouts were constructed using pCGEN-derived plasmids assembled via In-Fusion HD Cloning (Takara Bio). For each target gene, ~1-kb upstream and downstream flanking regions were amplified from the appropriate wild-type background and assembled with a hygromycin resistance cassette (from the pES6 vector) into a linearized pCGEN backbone. The assembled deletion constructs were propagated in *Escherichia coli* HST08 (Stellar competent cells, Takara Bio), verified via Oxford Nanopore full-plasmid sequencing (MicroSynth) and transformed into *Agrobacterium tumefaciens* strain AGL1. *Agrobacterium*-mediated transformation of *Z. tritici* was performed as described in Meile et al.^24^. Briefly, induced *A. tumefaciens* and *Z. tritici* cells were co-cultivated on induction medium for 3 d at 18 °C, and then transferred to selective media (cefotaxime plus hygromycin B or geneticin) to recover transformants. Single colonies were purified, and deletion mutants were confirmed by long-read sequencing of the targeted locus and screened for single-copy insertions by quantitative PCR using hygromycin-specific primers designed in Alassimone et al.^75^. The primer sequences are provided in Supplementary Table 10.

To further validate the causal role of two pathogenicity-related genes, ZtIPO323_003260 (Simano, a predicted effector) and ZtIPO323_103700 (Arobase, unknown function), we generated independent ectopic complementation lines. Complementation plasmids were constructed by amplifying the full-length coding sequence and native regulatory regions (~2 kb upstream and downstream) from ST99CH_1A5, cloned into the pCGEN vector and transformed into the corresponding knockout line. Three independent complemented lines per gene were recovered and validated by PCR and sequencing. Complemented strains, along with wild-type and knockout lines, were subjected to the same infection assay described above to evaluate phenotypic rescue.

Infection assays of the wild type, independent knockout (*n* = 2) and complementation (*n* = 3) lines were performed following the procedure described in Alassimone et al.^75^. Briefly, 17-day-old plants from each cultivar were inoculated with 20 ml of a spore suspension at 10^7^ spores per millilitre of water amended with 0.1% Tween 20. The plants were placed in the greenhouse, and the second leaf was harvested 21 days post-infection. The leaves were mounted on paper sheets and scanned (CanoScan LiDE 240). The PLACL was quantified with leaf-toolkit v.0.1.0 (ref. ^76^). The statistical comparisons of virulence level (PLACL) distributions were performed using paired Wilcoxon tests using each corresponding wild-type strain as the reference group with the R package ggpubr v.0.6.2 (ref. ^77^).

### Reporting summary

Further information on research design is available in the Nature Portfolio Reporting Summary linked to this article.

## Supplementary information

Supplementary Information Supplementary Figs. 1 and 2.

Reporting Summary

Supplementary Tables Supplementary Table 1: Metadata of the *Z. tritici* isolates in this study, including the isolate name, collection, host, plot ID, geographical sampling site and sampling dates, fungicide treatments, possible alternative names used in other publications and the NCBI Bioproject corresponding to the sequencing data, present in the final genomic dataset. Supplementary Table 2: Microsatellite primers used for clone correction and obtained from ref. ^62^. Supplementary Table 3: Allele scoring results from microsatellite analyses. Supplementary Table 4: Microsatellite loci summary statistics. Supplementary Table 5: Global statistics for the uncorrected dataset per collection time point. Supplementary Table 6: Global statistics for the uncorrected dataset per host cultivar. Supplementary Table 7: Analysis of molecular variance showing partitioning of genetic variation within and among host cultivars and collection time points. Supplementary Table 8: Number of biallelic SNPs tested per GWAS used to calculate Bonferroni thresholds. Supplementary Table 9: GWAS-identified loci in the reference genome annotation IPO323. Supplementary Table 10: Primers and plasmids used to generate knockout and complementation lines.

Supplementary Data 1 Raw and statistical source data for Supplementary Fig. 1.

Supplementary Data 2 Raw and statistical source data for Supplementary Fig. 2.

## Source data

Source Data Fig. 1 Statistical source data.

Source Data Fig. 2 Raw and statistical source data.

Source Data Fig. 3 Raw and statistical source data.

Source Data Extended Data Fig. 1 Raw and statistical source data.

Source Data Extended Data Fig. 2 Raw and statistical source data.

Source Data Extended Data Fig. 3 Raw and statistical source data.

Source Data Extended Data Fig. 4 Raw and statistical source data.

Source Data Extended Data Fig. 5 Raw and statistical source data.

Source Data Extended Data Fig. 6 Raw and statistical source data.

Source Data Extended Data Fig. 7 Raw and statistical source data.

Source Data Extended Data Fig. 8 Raw and statistical source data.

Source Data Extended Data Fig. 9 Raw and statistical source data.

Source Data Extended Data Fig. 10 Raw and statistical source data.

## Data Availability

All sequencing data are available from the NCBI Sequence Read Archive under BioProject numbers PRJNA1116850 and PRJNA596434 (ref. ^61^). All data for GHA analysis are available via Zenodo at https://doi.org/10.5281/zenodo.11263000 (ref. ^78^). Plasmid maps used for generating the knockout lines are available via GitHub at https://github.com/lorraincecile/GHA_Ztritici. Source data are provided with this paper.

## Code availability

The customized scripts generated for this study are available via GitHub at https://github.com/lorraincecile/GHA_Ztritici.
