# Supplementary material for: Genome–host association mapping reveals wheat pathogen genes involved in host specialization
Source: Nat Plants. 2026 Apr 15;12(4):791–801. doi: 10.1038/s41477-026-02269-w (PMC13652737; doi:10.1038/s41477-026-02269-w)
Supplement: Supplementary file 1 [file 41477_2026_2269_MOESM1_ESM.pdf]

nature plants

Supplementary information

https://doi.org/10.1038/s41477-026-02269-w

Genome–host association mapping reveals wheat pathogen genes involved in host specialization

In the format provided by the authors and unedited

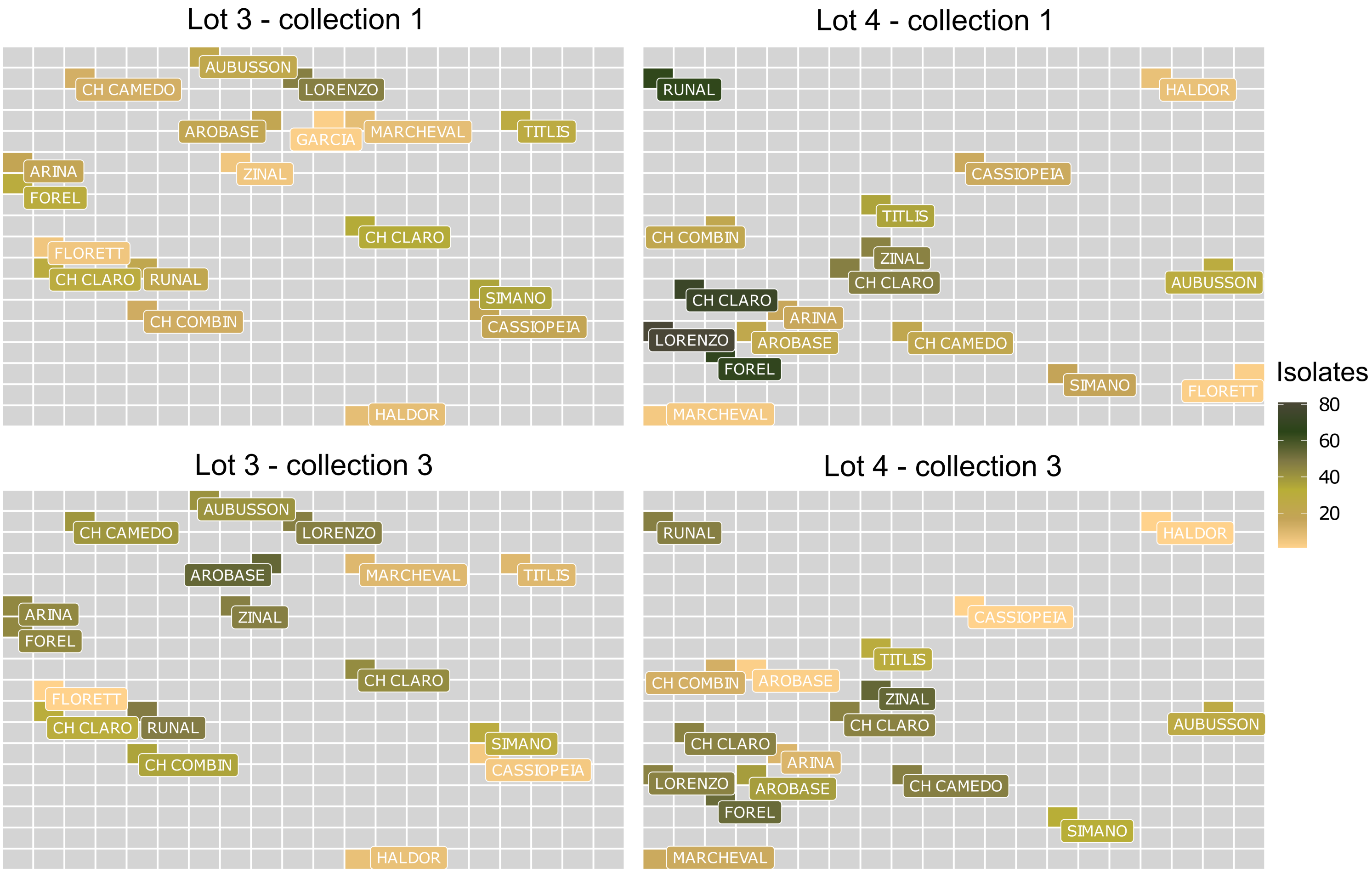

Supplementary Figure 1: Schematic map of the sampling site at the Field Phenotyping Platform of Eschikon Field Station of the ETH Zurich. The top panel represents the collection harvested in May (collection 1) on the two replicated fields “lot 3” and “lot 4”. The bottom panel represents the collection harvest in July (collection 3). Each plot is labeled with the cultivar name and colored according to the number of isolates.

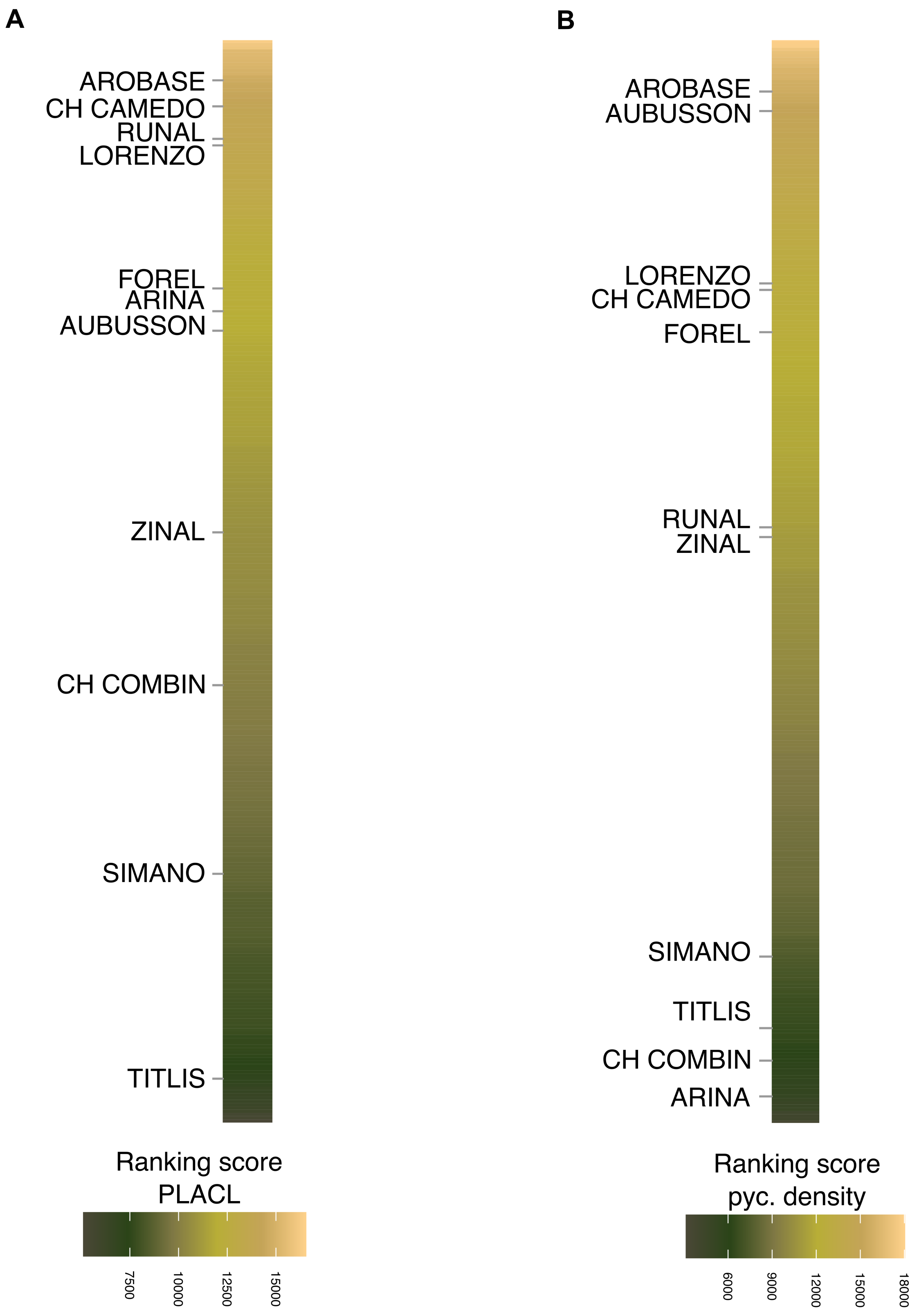

Supplementary Figure 2: Wheat cultivars quantitative resistance rankings from Karisto et al. (2018)[30] based on A) percentage of leaves area covered by lesions (PLACL) and B) pycnidia density per leaf. The ranking values were calculated over 335 wheat cultivars using the mean rank values derived from the Kruskal-Wallis test applied to 21,420 individually phenotyped leaves, as described in Karisto et al. (2018)[30]. Each leaf was ranked from most resistant (lowest rank) to most susceptible (highest rank), and the mean rank per cultivar was used as an overall measure of susceptibility. Cultivars included in our study are marked along the ranking scale. The cultivars included in our study are marked on the ranking scale. The colored scale represents the most resistant cultivars in green to the most susceptible cultivars in yellow.
