# Supplementary material for: Genome–host association mapping reveals wheat pathogen genes involved in host specialization
Source: Nat Plants. 2026 Apr 15;12(4):791–801. doi: 10.1038/s41477-026-02269-w (PMC13652737; doi:10.1038/s41477-026-02269-w)
Supplement: Supplementary file 2 [file 41477_2026_2269_MOESM2_ESM.pdf]

nature portfolio

Corresponding author(s): Cecile Lorrain

Last updated by author(s): Dec 2, 2025

Reporting Summary

Nature Portfolio wishes to improve the reproducibility of the work that we publish. This form provides structure for consistency and transparency in reporting. For further information on Nature Portfolio policies, see our Editorial Policies and the Editorial Policy Checklist.

Statistics

For all statistical analyses, confirm that the following items are present in the figure legend, table legend, main text, or Methods section.

n/a	Confirmed	
☐	☒	The exact sample size (n) for each experimental group/condition, given as a discrete number and unit of measurement
☐	☒	A statement on whether measurements were taken from distinct samples or whether the same sample was measured repeatedly
☐	☒	The statistical test(s) used AND whether they are one- or two-sided<br>*Only common tests should be described solely by name; describe more complex techniques in the Methods section.*
☐	☒	A description of all covariates tested
☐	☒	A description of any assumptions or corrections, such as tests of normality and adjustment for multiple comparisons
☐	☒	A full description of the statistical parameters including central tendency (e.g. means) or other basic estimates (e.g. regression coefficient) AND variation (e.g. standard deviation) or associated estimates of uncertainty (e.g. confidence intervals)
☒	☐	For null hypothesis testing, the test statistic (e.g. F, t, r) with confidence intervals, effect sizes, degrees of freedom and P value noted<br>*Give P values as exact values whenever suitable.*
☒	☐	For Bayesian analysis, information on the choice of priors and Markov chain Monte Carlo settings
☒	☐	For hierarchical and complex designs, identification of the appropriate level for tests and full reporting of outcomes
☒	☐	Estimates of effect sizes (e.g. Cohen's d, Pearson's r), indicating how they were calculated

Our web collection on statistics for biologists contains articles on many of the points above.

Software and code

Policy information about availability of computer code

Data collection	Data collection:<br>Fragman R package for microsatellite allele scoring.<br>3730 DNA Analyzer software (Applied Biosystems).<br>Leaf-toolkit 0.1.0 for lesion quantification.<br>Nanopore sequencing basecalling software (MicroSynth-provided).<br>CanoScan LiDE 240 scanning software.
Data analysis	The customized scripts and downstream analyses generated for this study are available in the following repositories: https://github.com/lorraincecile/GHA_Ztritici<br>Data analysis software<br>Trimmomatic v0.36<br>BWA v0.7.17<br>PICARD v2.23.8<br>GATK v4.1.2.0<br>VCFtools v0.1.16<br>bcftoolsv1.16<br>PLINK v1.9<br>LEA R package (sNMF)<br>poppr R package<br>Fragman R package<br>vcf2gwas v0.8.7

GEMMA
SnpEffv5.2
CLC Workbench 23.05 (BLAST)
R v4.x for all scripts.

For manuscripts utilizing custom algorithms or software that are central to the research but not yet described in published literature, software must be made available to editors and reviewers. We strongly encourage code deposition in a community repository (e.g. GitHub). See the Nature Portfolio guidelines for submitting code & software for further information.

Data

Policy information about availability of data

All manuscripts must include a data availability statement. This statement should provide the following information, where applicable:

- Accession codes, unique identifiers, or web links for publicly available datasets
- A description of any restrictions on data availability
- For clinical datasets or third party data, please ensure that the statement adheres to our policy

All sequencing data is available from the NCBI Sequence Read Archive, under the Bioproject number PRJNA1116850 and PRJNA59643457. All source data for GHA are available from the following repository: https://doi.org/10.5281/zenodo.17751192. Plasmid maps used for generating the knockout lines are available in the Github repository: https://github.com/lorraincecile/GHA_Ztritici.

Research involving human participants, their data, or biological material

Policy information about studies with human participants or human data. See also policy information about sex, gender (identity/presentation), and sexual orientation and race, ethnicity and racism.

Reporting on sex and gender	NA
Reporting on race, ethnicity, or other socially relevant groupings	NA
Population characteristics	NA
Recruitment	NA
Ethics oversight	NA

Note that full information on the approval of the study protocol must also be provided in the manuscript.

Field-specific reporting

Please select the one below that is the best fit for your research. If you are not sure, read the appropriate sections before making your selection.

☒ Life sciences ☐ Behavioural & social sciences ☐ Ecological, evolutionary & environmental sciences

For a reference copy of the document with all sections, see nature.com/documents/nr-reporting-summary-flat.pdf

Life sciences study design

All studies must disclose on these points even when the disclosure is negative.

Sample size	The sample sizes where determined based on the number of strains isolated and sequenced.
Data exclusions	No data were excluded from the analysis.
Replication	For gene candidate functional validation, we analyzed two independent deletion lines per gene and three independent ectopic complementation lines per gene.
Randomization	For GHA, the control group was composed of randomly subsampled strains from the alternative host, excluding those present in the test group.
Blinding	Blinding was not applicable in this study.

Reporting for specific materials, systems and methods

We require information from authors about some types of materials, experimental systems and methods used in many studies. Here, indicate whether each material, system or method listed is relevant to your study. If you are not sure if a list item applies to your research, read the appropriate section before selecting a response.

Materials & experimental systems

n/a	Involved in the study
☒	☐ Antibodies
☒	☐ Eukaryotic cell lines
☒	☐ Palaeontology and archaeology
☐	☒ Animals and other organisms
☒	☐ Clinical data
☒	☐ Dual use research of concern
☐	☒ Plants

Methods

n/a	Involved in the study
☒	☐ ChIP-seq
☒	☐ Flow cytometry
☒	☐ MRI-based neuroimaging

Animals and other research organisms

Policy information about studies involving animals; ARRIVE guidelines recommended for reporting animal research, and Sex and Gender in Research

Laboratory animals	NA
Wild animals	NA
Reporting on sex	NA
Field-collected samples	Fungal isolates were collected from a natural epidemic at the Field Phenotyping Platform of the Eschikon Field Station of ETH Zurich, Switzerland (coordinates 47.449°N, 8.682°E). Strains were isolated by streak-plating of individual cirri on yeast malt sucrose agar (YMA) amended with kanamycin (50ug/L). Single colonies were subsequently grown in yeast sucrose broth (YSB) to produce blastospores that were used for long-term storage
Ethics oversight	NA

Note that full information on the approval of the study protocol must also be provided in the manuscript.

Plants

Seed stocks	Wheat seeds were obtained from the GABI wheat panel, all commercial cultivars.
Novel plant genotypes	NA
Authentication	All wheat cultivars come from commercial varieties.
